# Two new species of parasitic copepods from the genera *Nothobomolochus* and *Unicolax* (Cyclopoida: Bomolochidae) from Australian waters

**DOI:** 10.7717/peerj.6858

**Published:** 2019-05-02

**Authors:** James P. Bernot, Geoffrey A. Boxshall

**Affiliations:** 1 Institute for Biomedical Sciences, George Washington University, Washington, DC, United States of America; 2 Department of Life Sciences, The Natural History Museum, London, United Kingdom

**Keywords:** Parasite, Bomolochidae, Copepod, *Unicolax*, *Nothobomolochus*, Copepoda, Fish, Hosts, Biogeography

## Abstract

A 2016 collaborative survey of commercial fish parasites in Moreton Bay, Queensland, Australia led to the discovery of two new species of parasitic copepods belonging to the family Bomolochidae. Females of *Nothobomolochus johndaveorum* n. sp. were found attached to the gill filaments of *Gerres subfasciatus* and *Gerres oyena*. The new species most closely resembles *N. leiognathicola* and *N. quadriceros*. All 3 species possess 3 modified setae on the first antennulary segment that are approximately the same length and have a robust seta on the second antennulary segment adjacent to the 3 modified setae giving a superficial appearance of 4 modified setae on the antennule. The new species can be distinguished from these two species in its possession of longer inner setae on the first two endopodal segments of leg 4: the seta on endopodal segment 1 extends past the midline of the distal segment in the new species vs to the proximal margin of the distal segment in the other two species, and the seta on segment 2 extends well beyond the distal margin of the endopod in the new species vs just to the margin in the other two species. Females and males of *Unicolax longicrus* n. sp. were found in the nasal sinuses of *Sillago maculata* and *Sillago ciliata*. The new species differs from 6 of its 7 congeners in having a leg 4 exopod formula of II, I, 4 rather than II, I, 3 or II, I, 5. The new species resembles *U. anonymous* in this feature, but differs in its possession of a leg 5 that is relatively longer and less wide, and, whereas *U. anonymous* possesses inner and outer distal spines on leg 5 that are approximately the same length, those of the new species are relatively longer and asymmetrical. *Unicolax longicrus* n. sp. is unique among its congeners in its possession of a leg 4 with highly elongated endopodal segments 2 and 3, from which its name is derived. In addition to describing the two new species, host and locality reports for all species of *Nothobomolochus* and *Unicolax* are reviewed.

## Introduction

As part of a concerted effort to survey parasites of commercial fish of Moreton Bay, Queensland, Australia, a diversity of fish species were examined for parasitic copepods. Two new species of copepods belonging to the family Bomolochidae Claus, 1875 collected from Australian teleosts during this survey are described below: one belonging to the gill-inhabiting genus *Nothobomolochus* Vervoort, 1962 and the other the nostril-inhabiting genus *Unicolax* Cressey & Cressey, 1980. In addition to describing these two new species, we review all host and locality reports for each species of *Nothobomolochus* and *Unicolax*.

## Materials & Methods

Fish were collected by tunnel net or rod-and-reel in Moreton Bay, Queensland, Australia in January and June of 2016 under permit 187264 from the Queensland Department of Agriculture, Fisheries, and Forestry following the guidelines of the Animal Welfare Unit at the University of Queensland (approval number SBS/248/15/ABRS/ARC). Fish examined consisted of 4 specimens of *Gerres oyena* (Forsskål, 1775), 4 specimens of *Gerres subfasciatus* Cuvier, 1830, 1 specimen of *Sillago ciliata* Cuvier, 1829, and 1 specimen of *Sillago maculata* Quoy & Gaimard, 1824. The body surface, gill arches, and nasal passages were examined for parasitic copepods using a dissecting microscope. Copepods were preserved in 70% ethanol at the time of collection. Specimens were cleared in lactic acid for at least 3 h prior to examination in glass cavity slides using a Leica dissecting microscope. When necessary, appendages were dissected using tungsten wire needles that had been electrolytically sharpened in saturated potassium hydroxide following standard protocols. Observations and drawings were made on an Olympus BX51 compound microscope equipped with differential interference contrast (DIC) and a drawing tube. Measurements were made on the same microscope using an ocular reticule. Measurements are given in micrometers and are presented as the minimum and maximum, followed in parentheses by the mean, standard deviation, and number of specimens measured. Setation formulae are given from proximal to distal segment, separated by semicolons, with Roman numerals indicating spines and Arabic numerals indicating setae; aesthetasks are indicated with ae. Appendage terminology follows Huys & Boxshall (1991). Fish taxonomy follows Betancur et al. (2017) for classification above the family level, and Fishbase (Froese & Pauly, 2018) for family level and below. Museum abbreviations used are as follows: QM, Queensland Museum, South Brisbane, Australia; NHMUK, The Natural History Museum, Department of Life Sciences, London, UK; USNM, National Museum of Natural History, Smithsonian Institution, Washington, D.C., USA. The electronic version of this article in Portable Document Format (PDF) will represent a published work according to the International Commission on Zoological Nomenclature (ICZN), and hence the new names contained in the electronic version are effectively published under that Code from the electronic edition alone (International Commission on Zoological Nomenclature, 2012). This published work and the nomenclatural acts it contains have been registered in ZooBank, the online registration system for the ICZN. The ZooBank LSIDs (Life Science Identifiers) can be resolved and the associated information viewed through any standard web browser by appending the LSID to the prefix http://zoobank.org/. The LSID for this publication is: urn:lsid:zoobank.org:pub:E8663AB9-EF47-4382-8096-C45B10A879A7. The online version of this work is archived and available from the following digital repositories: PeerJ, PubMed Central, and CLOCKSS.

## Results

**Table:** 

**Figure 1:**
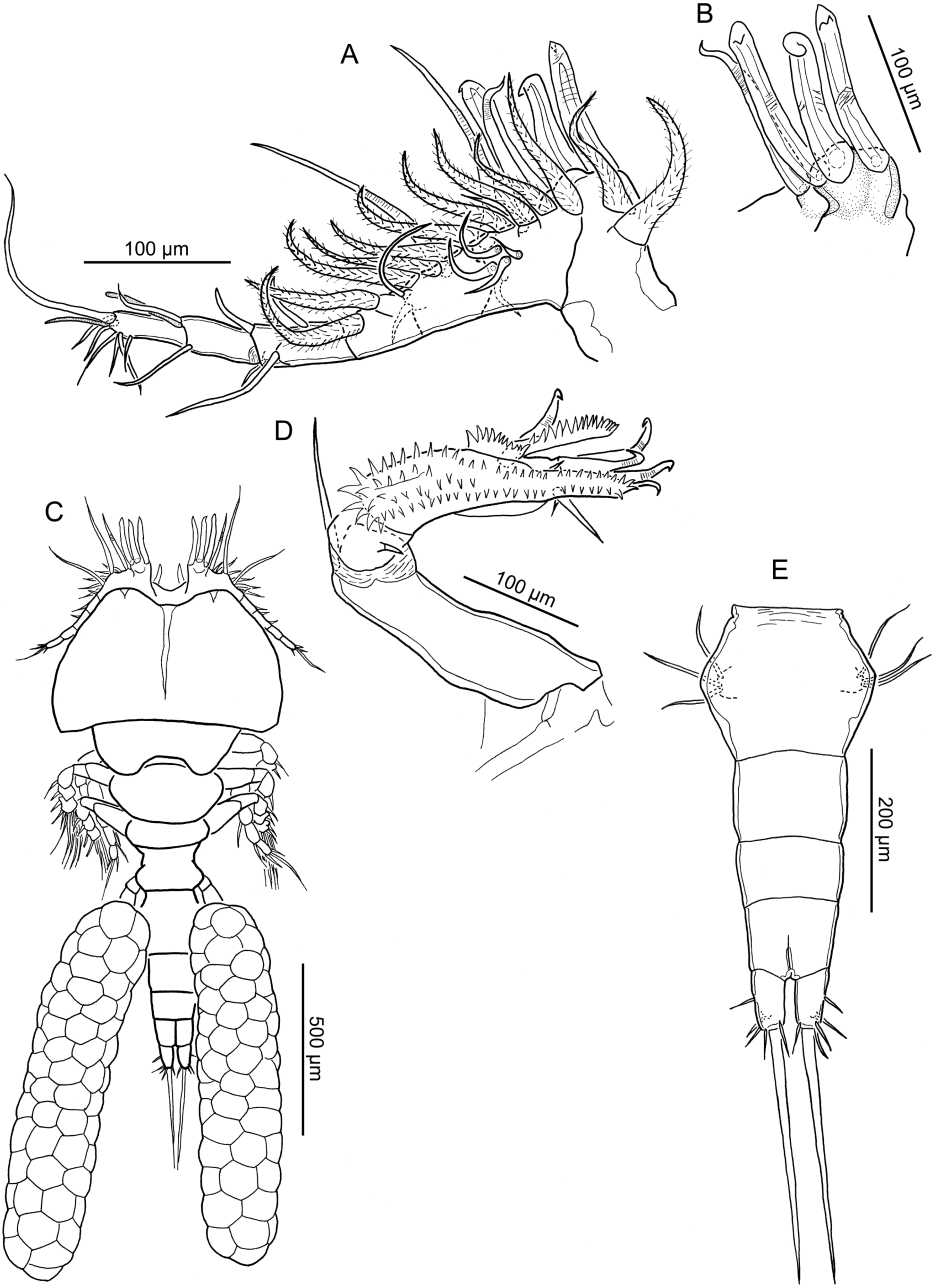
Line drawings of *Nothobomolochus johndaveorum* n. sp. (A) Antennule, ventral view (dissected paratype). (B) Pedestal of first antennulary segment and modified adjacent seta, dorsal view (dissected paratype). (C) Habitus, dorsal view (holotype QM W29438). (D) Antenna, ventral view (dissected paratype). (E) Urosome, ventral view (holotype QM W29438). Drawing credit: James P. Bernot.

**Figure 2:**
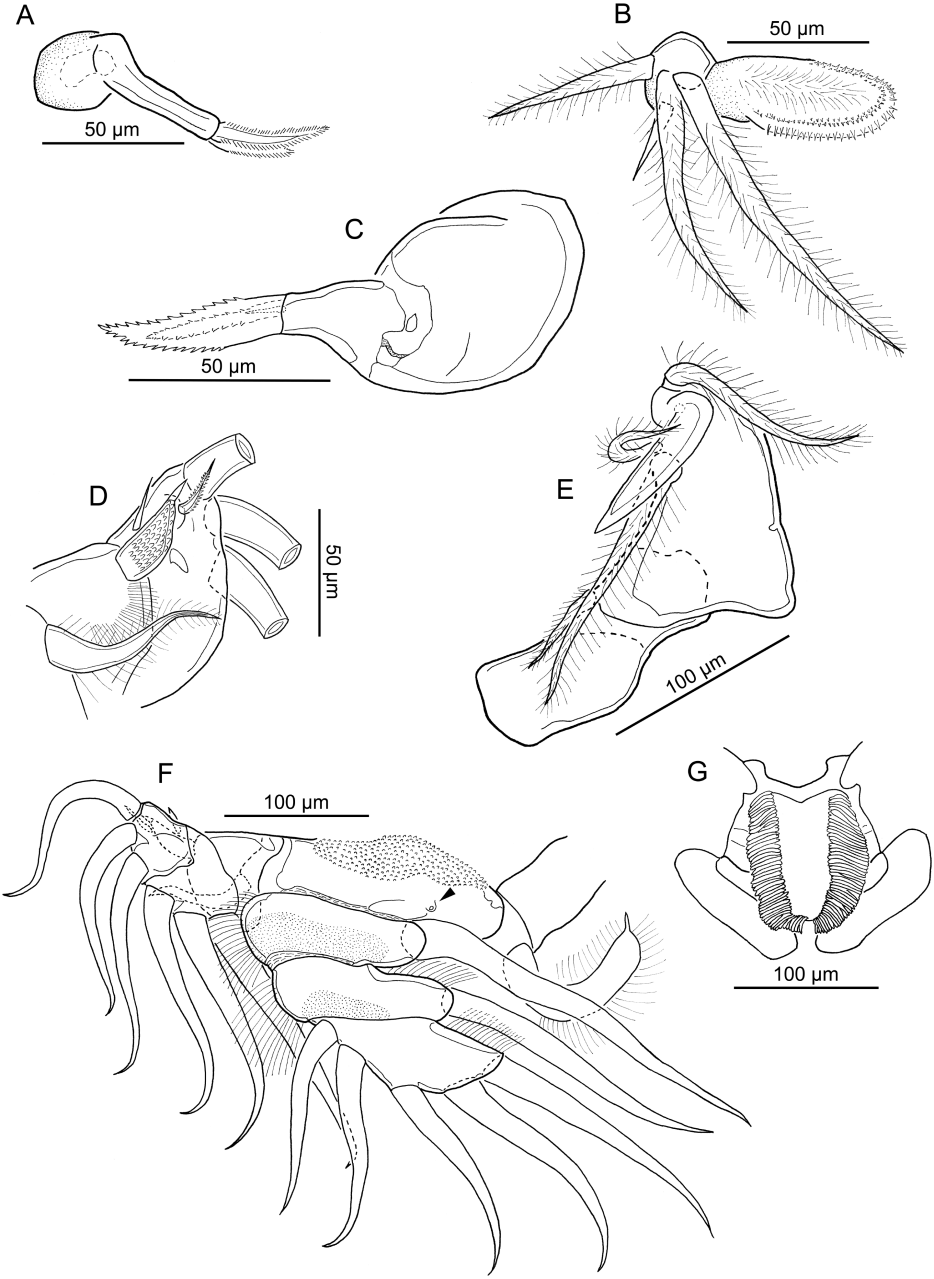
Line drawings of *Nothobomolochus johndaveorum* n. sp. (A) Mandible, ventral view (dissected paratype). (B) Maxillule and paragnath, ventral view (dissected paratype). (C) Maxilla, ventral view (dissected paratype). (D) Outer spines on leg 1 exopod, dorsal view (dissected paratype). (E) Maxilliped, ventral view (dissected paratype). (F) Leg 1, ventral view (dissected paratype), plumosity on setae not illustrated; arrowhead indicating inner seta represented by small rounded tubercle. (G) Intercoxal sclerite of leg 1, ventral view (holotype QM W29438). Drawing credit: James P. Bernot.

**Figure 3:**
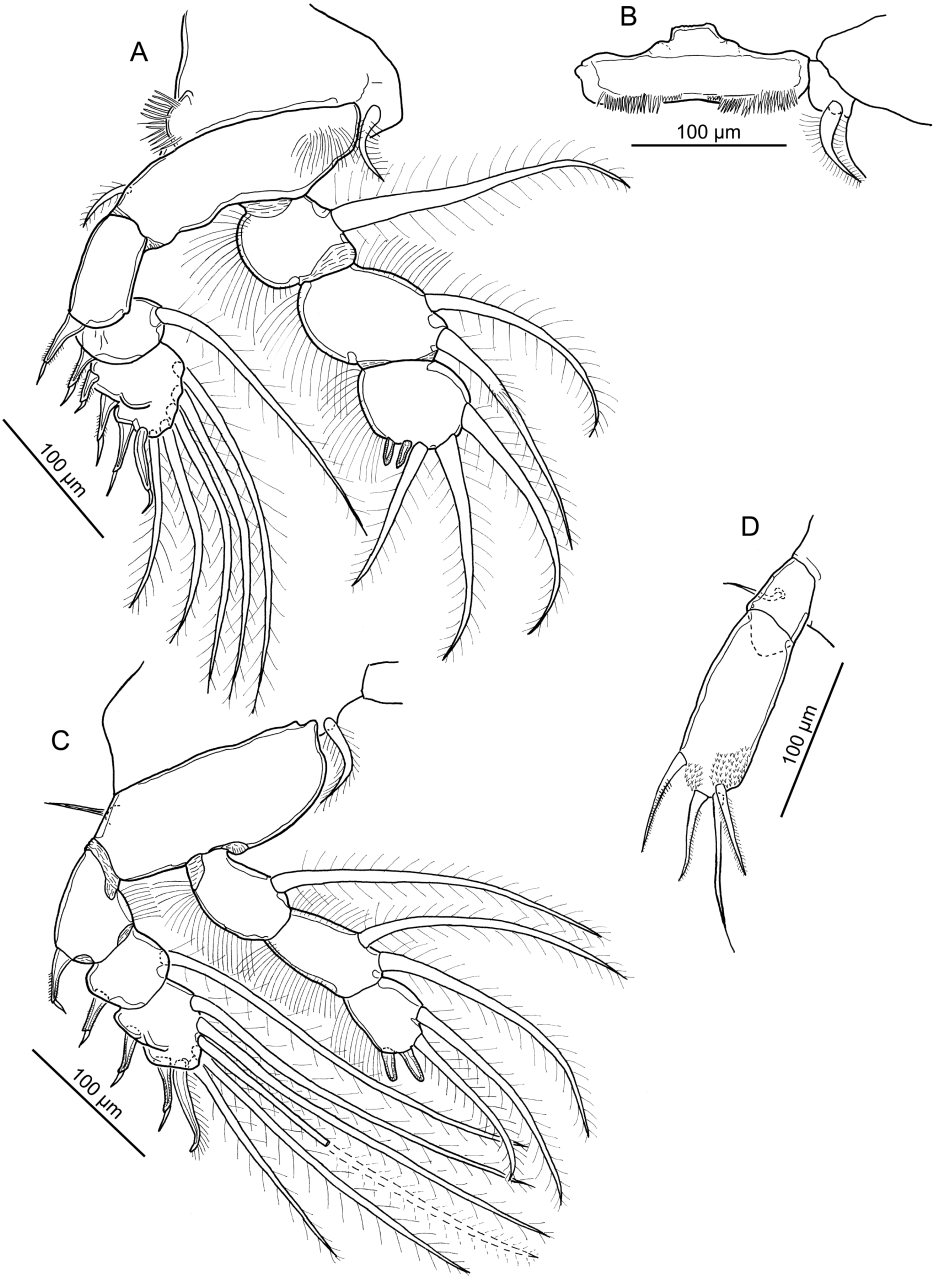
Line drawings of *Nothobomolochus johndaveorum* n. sp. (A) Leg 2, ventral view (dissected paratype). (B) Intercoxal sclerite of leg 2, ventral view (dissected paratype). (C) Leg 3, ventral view (dissected paratype). (D) Leg 5, ventral view (dissected paratype). Drawing credit: James P. Bernot.

**Figure 4:**
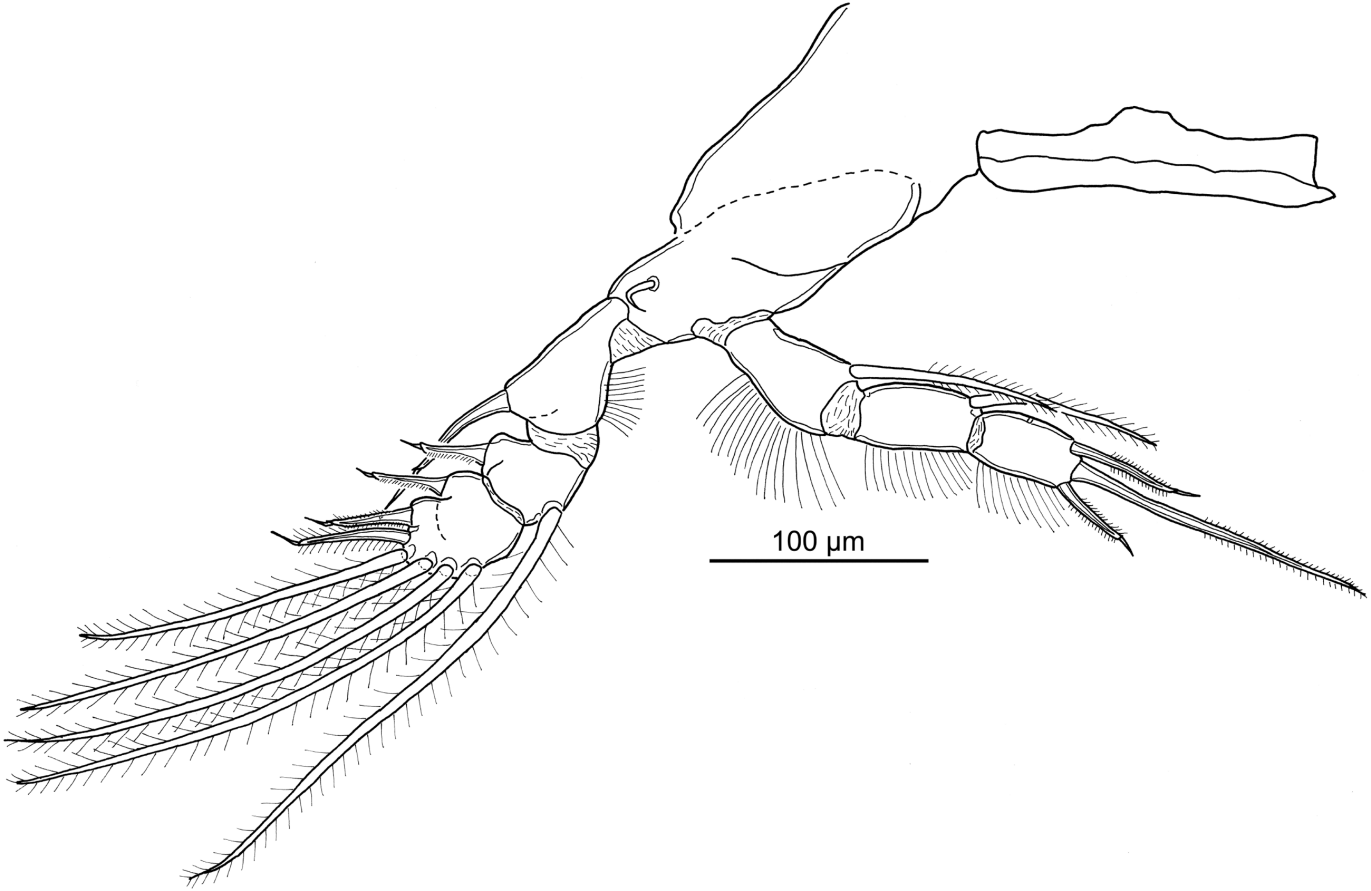
Line drawing of *Nothobomolochus johndaveorum* n. sp. Leg 4 and intercoxal sclerite, dorsal view (dissected paratype). Drawing credit: James P. Bernot.

Family Bomolochidae Claus, 1875
Genus *Nothobomolochus*Vervoort, 1962
*Nothobomolochus johndaveorum* n. sp.
(Figs. 1–4).

*LSID:* urn:lsid:zoobank.org:act:D5F768DE-686A-4B00-B241-8BBA061BF4A9

*Type-host*: *Gerres subfasciatus* Cuvier, 1830 (Gerreiformes: Gerridae).

*Other host: Gerres oyena* (Forsskål, 1775) (Gerreiformes: Gerridae).

*Type-locality*: Moreton Bay, Queensland, Australia (27°22′S, 153°13′E).

*Site*: Branchial chamber attached to gill lamellae.

*Type-material*: Holotype female (QM W29438) and 11 female paratypes (2 paratypes QM W29439; 4 paratypes NHMUK 2018.194–2018.197; 4 paratypes USNM 1532298–1532300), 1 fully dissected female paratype not deposited.

*Etymology*: This species is named in honor of John Page and Dave Thompson for their generosity in providing us with host specimens.

Adult female.

Body (Fig. 1C) 1,000–1,642 (1,371 ± 172; *n* = 9) long, measured along midline from frontal margin of rostrum to posterior margin of caudal rami excluding caudal setae; greatest width 602–768 (684 ± 48; 11) at posterior of dorsal cephalothoracic shield. Prosome 648–994 (815 ± 101; 10) long by 602–768 (684 ± 48; 11) wide, comprising broad cephalothorax and 3 free pedigerous somites. Urosome 467–647 (555 ± 62; 10) long by 196–241 (221 ± 15; 10) wide, comprising 5th pedigerous somite, genital double-somite, and 3 free abdominal somites. Genital double-somite (Fig. 1E) bearing paired genital apertures dorso-laterally. Abdominal somites unornamented; anal somite with anal slit deeply incised; bearing caudal rami. Caudal rami longer than wide, bearing principal seta and 5 small setae. Egg sacs elongate, multiseriate. Swimming leg armature summarized in Table 1.

**Table 1:** *Nothobomolochus johndaveorum* n. sp. swimming leg armature.

	Coxa	Basis	Exopod	Endopod
Leg 1	0-1	1-I	I-0; II, 6	0-1; 0-1; 5
Leg 2	0-1	1-0	I-0; I-1; IV, 5	0-1; 0-2; II, 3
Leg 3	0-1	1-0	I-0; I-1; II, I, 5	0-1; 0-2; II, 2
Leg 4	0-0	1-0	I-0; I-1; III, 4	0-1; 0-1; III

Antennule (Fig. 1A) indistinctly 7-segmented; first segment heavily sclerotized at base; second to fourth segments partially fused; 3 distal segments cylindrical. First segment with frontally-directed pedestal bearing 3 modified, spine-like setae (Fig. 1B) plus 2 hirsute setae proximally. Compound segments 2, 3, and 4 bearing in total: 10 hirsute setae arrayed along anterior margin, one proximal claw-like naked seta (Fig. 1B) and 2 conspicuously elongate naked setae on anterior margin, and 2 simple naked setae plus cluster of 4 naked setae on ventral surface; 2 short setae present on dorsal surface of segment. Distal 3 segments with the following setal formula: 4; 2 + 1 ae; 7 + 1 ae.

Antenna (Fig. 1D) uniramous, 3-segmented, comprising elongate coxobasis bearing long naked seta, short first endopodal segment bearing short seta, and heavily armed compound distal segment. Distal segment ornamented with 4 irregular rows of spinules, 2 rows of spinules extending onto elongate distal process; bearing one pectinate process extending distally and one medial, fan-like pectinate process; distal armature comprising 4 claw-like setae and 4 naked setae of unequal lengths.

Mandible (Fig. 2A) bearing two spinulate blades; ventral blade slightly longer than dorsal. Paragnath (Fig. 2B) ovoid, ornamented with multiple rows of small tooth-like spinules on margin and hairs along midline. Maxillule (Fig. 2B) consisting of rounded lobe with 3 hirsute setae and 1 small naked seta. Maxilla (Fig. 2C) indistinctly 2-segmented; proximal segment unarmed; distal segment with 2 unequal spinulate processes and 1 dorsal, small naked seta. Maxilliped (Fig. 2E) 3-segmented, comprising long syncoxa armed with one hirsute seta; middle segment tapering distally, armed with 2 unequal hirsute setae on medial margin; distal segment incorporated into claw and bearing hirsute seta; claw simple, lacking accessory process.

Leg 1 (Fig. 2F) biramous, with flattened lamellate rami; members of leg pair joined by intercoxal sclerite (Fig. 2G) bearing lingulate process ornamented with elongate spinules on lateral margins. Coxa with inflated plumose seta, with blunt-apex drawn out into elongate tip. Basis ornamented with patch of rounded spinules proximally, outer plumose seta (Figs. 2D and 2F), and inner seta reduced to small rounded tubercle (arrowhead in Fig. 2F). Exopod (Figs. 2D and 2F) 2-segmented; proximal segment armed with large spine on dorsal surface ornamented with lingulate spinules and narrow tip; compound distal segment formed by fusion of segments 2 and 3, bearing 6 plumose setae (only 3 shown in Fig. 2D; plumosity not figured in Figs. 2D and 2F), 1 naked spine and 1 serrated spine on dorsal surface (Fig. 2D). Secondary cuticular thickenings present on ventral surface of compound distal segment, not indicative of original segmental articulations. Endopod 3-segmented; all segments flattened and expanded transversely; first and second segments each with inner plumose seta and ornamented with patch of small spinules on ventral surface and hair-like setules on outer margin; second segment with additional hair-like setules along inner margin; third segment armed with 5 plumose setae and with hair-like setules along inner margin.

Leg 2 (Fig. 3A) biramous; intercoxal sclerite (Fig. 3B) ornamented with 2 lateral fields of spinules. Coxa with inner plumose seta and outer swelling ornamented with elongate, blunt-tipped spinules. Basis with outer plumose seta and inner patch of hair-like setules proximally. Exopod 3-segmented, with setal formula: I-0; I-1; IV, 5. All spines bearing subterminal flagellum, and all but terminal 2 spines with bilateral spinulation. Endopod 3-segmented; first segment with plumose inner seta and hair-like setules on outer margin; second segment with 2 plumose inner setae and hair-like setules on inner and outer margins; third segment with 2 short, bilaterally spinulate spines and 3 plumose setae, and ornamented with hair-like setules on outer margin.

Leg 3 (Fig. 3C) biramous, with unornamented intercoxal sclerite. Coxa with inner plumose seta. Basis with outer naked seta on raised base. Exopod 3-segmented; first segment with outer spine and hair-like setules on inner margin; second segment with outer spine and inner seta; third segment with formula: II, I, 5. All outer spines with bilateral spinulation and subterminal flagellum. Terminal spine with spinulation on inner margin only and lacking flagellum. Endopod 3-segmented; first segment with plumose inner seta and hair-like setules along outer margin; second segment with 2 plumose setae and setules along inner and outer margins; third segment with 2 inner setae plus 2 spines bearing fine spinulation bilaterally, and ornamented with hair-like setules along outer margin.

Leg 4 (Fig. 4) biramous, with broad unornamented intercoxal sclerite. Coxa lacking inner seta. Basis with naked outer seta on slightly raised base. Exopod 3-segmented; first segment with long outer spine bearing elongate subterminal flagellum and ornamented with hair-like setules along inner margin of segment; second segment bearing plumose seta and outer spine ornamented with flagellum and unilateral spinulation; third segment with setal formula: III, 4; all spines with flagellum; proximal spine with unilateral spinulation; distal 2 spines with bilateral spinulation. Endopod 3-segmented; all segments ornamented with row of long hair-like setules along outer margin: first segment bearing plumose seta extending just beyond middle of third segment; second segment with inner plumose seta extending beyond tip of ramus, about to middle of outer distal spine; third segment with 3 bilaterally-spinulate spines distally; inner and outer distal spines each with flagellate tip; inner distal spine 30% longer than outer but only 40% as long as apical spine.

Leg 5 (Fig. 3D) 2-segmented: protopodal segment with naked outer seta; exopodal segment with 4 setae; outer 2 setae each with unilateral spinulation; terminal seta naked, inner seta with bilateral spinulation; terminal seta markedly longer than other elements but just shorter than segment. Leg 6 (Fig. 1E) represented by 3 setae on raised base near oviduct opening.

### Remarks

The genus *Nothobomolochus* currently comprises 39 valid species (Walter & Boxshall, 2019), and the most recent key to species was provided by El-Rashidy & Boxshall (2014), although this does not include *N. ilhoikimi* Venmathi Maran et al., 2014. *Nothobomolochus johndaveorum* n. sp. can be readily distinguished from *N. fradei* Marques, 1965, *N. ilhoikimi*, *N. lateolabracis* (Yamaguti & Yamasu, 1959) Vervoort, 1962, *N. lizae* Ho and Lin, 2005, and *N. sagaxi* Avdeev, 1986 in its possession of 3, rather than 2, apical elements on the distal endopodal segment of leg 4. The new species differs from *N. cornutus* (Claus, 1864) Vervoort, 1962, *N. cresseyi* Timi & Sardella, 1997, *N. cypseluri* (Yamaguti, 1953) Vervoort, 1962, *N. exocoeti* Avdeev, 1978, *N. gibber* (Shiino, 1957) Vervoort, 1962, *N. monodi* El-Rashidy & Boxshall, 2014, *N. oxyporhamphi* Avdeev, 1977, *N. paruchini* Avdeev, 1978, *N. scomberesocis* (Krøyer, 1863) Vervoort, 1962, *N. teres* (Wilson, 1911) Pillai, 1967, and *N. trichiuri* Pillai & Natarajan, 1977 in possessing 3 modified setae on the pedestal on the first antennulary segment that are of approximately the same length, rather than possessing a proximal element at least 20–60% shorter than the 2 more distal processes. The new species can be differentiated from *N. epulus* Vervoort, 1962, *N. gazzae* (Shen, 1957) Vervoort, 1969, *N. kanagurta* (Pillai, 1965) Cressey & Cressey, 1980, *N. longisaccus* Ho and Lin, 2005, *N. neomediterraneus* El-Rashidy and Boxshall, 2001, *N. ovalis* Avdeev, 1977, and *N. sigani* Hameed & Kumar, 1988 in its lack of an accessory process on the female maxilliped claw.

The new species differs from *N. multispinosus* (Gnanamuthu, 1949) Vervoort, 1962, *N. triceros* (Bassett-Smith, 1898) Vervoort, 1962, and *N. vervoorti* Avdeev, 1986 in its possession of modified setae on the antennulary pedestal that are less than 1/3 the length of the cephalothorax, rather than greater than 1/3 the length. *Nothobomolochus johndaveorum* n. sp. further differs from the former two species in its possession of caudal rami that are shorter than the anal somite. The new species is readily distinguished from *N. atlanticus* Avdeev, 1978, *N. chilensis* Avdeev, 1974, *N. elegans* Avdeev, 1977, *N. marginatus* Avdeev, 1986, and *N. pulicatensis* Kaliyamurthy, 1990 in having a leg 4 distal exopod formula of II, I, 4 rather than II, I, 5.

*Nothobomolochus johndaveorum* n. sp. differs from *N. saetiger* (Wilson, 1911) Vervoort, 1962 in its possession of caudal rami that are longer than wide. It can be differentiated from *N. denticulatus* (Bassett-Smith, 1898) Vervoort, 1962, *N. digitatus* Cressey, 1970, *N. gerresi* Pillai, 1973, and *N. thambus* Ho, Do & Kasahara, 1983 in its possession of a robust, modified seta on the second antennulary segment adjacent to the 3 modified setae on the pedestal of the first segment, that gives the appearance of a fourth modified process, rather than a typical unmodified seta. The new species further differs from these 4 species in possessing a much shorter third pedigerous somite rather than a swollen third pedigerous somite that completely, or nearly completely, overlaps the fourth pedigerous somite, concealing it in dorsal view.

The new species closely resembles *N. leiognathicola* El-Rashidy & Boxshall, 2014 and *N. quadriceros* Pillai, 1973 in that all species possess a robust, modified seta on the second antennulary segment adjacent to the 3 modified setae on the first segment so that, superficially, they appear to have 4 modified setae on the antennule. It can be differentiated from *N. leiognathicola* in its possession of an outer element on the distal endopodal segment of leg 4 that is 3/4 the length of the inner element, rather than $ \frac{1}{2} $ the length as in *N. leiognathicola*. The new species also possesses mandibular blades that are less asymmetrical: the shorter blade is 3/4 the length of the longer blade vs less than $ \frac{1}{2} $ the length in *N. leiognathicola*. In addition, the inner setae on the first 2 endopodal segments of leg 4 are much longer in the new species: the seta on endopodal segment 1 extends past the midline of the distal segment in the new species vs just past the margin of segment 2 in *N. leiognathicola*, and the seta on segment 2 extends well beyond the distal margin of the endopod in the new species vs just to the margin in *N. leiognathicola*. *Nothobomolochus johndaveorum* n. sp. is most similar to *N. quadriceros* but differs in the number of rows of spinules along the distal segment of the antenna. Whereas the new species possesses 4 rows of spinules, *N. quadriceros* as figured by Pillai (1973) possesses 9 rows of spinules. The new species can also be distinguished from *N. quadriceros* as figured by Pillai (1973) in the lengths of setal elements on leg 4: the outer spine on segment 2 of the exopod is relatively longer in the new species, extending past the midpoint of the third exopodal segment rather than just past the distal margin of the second exopodal segment; likewise the seta of endopodal segment 1 is relatively longer in the new species, extending past the midpoint of the third endopodal segment rather than to the distal margin of the second segment; and the distal endopodal segment of the new species bears an inner setal element that is 30% longer than the outer setal element, rather than approximately the same length.

One other species of *Nothobomolochus* has been reported parasitizing a species of *Gerres. Nothobomolochus gerresi* was described from *Gerres filamentosus* Cuvier by Pillai (1973) from Trivandrum (now Thiruvananthapuram), India. In addition to the characters noted above, the new species can be further distinguished from *N. gerresi* in its possession of a longer outer spine on the first exopodal segment of leg 4, which extends past the midpoint of the third endopodal segment rather than to the distal margin of the second segment, and its possession of 4, rather than 10, rows of spinules along the distal segment of the antenna.

**Table 2:** *Nothobomolochus* species hosts and localities. Records in bold indicate type hosts and localities.

**Species**	**Host**	**Host family**	**Host order**	**Locality**	**Marine Ecoregion**	**Source**
*N. atlanticus* Avdeev, 1978	***Exocoetus volitans***	Exocoetidae	Beloniformes	**Tropical Atlantic**	Tropical Atlantic	Avdeev (1978)
*N. chilensis* Avdeev, 1974	***Scomberesox saurus***	Belonidae	Beloniformes	**SE Pacific Ocean**	Eastern Indo Pacific	Avdeev (1974)
	*Cheilopogon furcatus*	Exocoetidae	Beloniformes	Tropical Atlantic	Tropical Atlantic	Avdeev (1978)
	Exocoetidae	Exocoetidae	Beloniformes	Gulf of Carpentaria, Australia	Central Indo Pacific	Avdeev (1977)
	Exocoetidae	Exocoetidae	Beloniformes	Japan	Temperate Northern Pacific	Avdeev (1977)
*N. cornutus* (Claus, 1864) Vervoort, 1962	***Luvarus imperialis***	Luvaridae	Acanthuriformes	**Messina Straits, Italy**	Temperate Northern Atlantic	Claus (1864)
*N. cresseyi* Timi & Sardella, 1997	***Engraulis anchoita***	Engraulidae	Clupeiformes	**Argentina**	Temperate South American	Timi & Sardella (1997)
*N. cypseluri* (Yamaguti, 1953) Vervoort, 1962	***Cheilopogon agoo***	Exocoetidae	Beloniformes	**Mie Prefecture, Japan**	Temperate Northern Pacific	Yamaguti (1953)
	*Cheilopogon cyanopterus*	Exocoetidae	Beloniformes	Northern Indian Ocean	Western Indo Pacific	Avdeev (1977)
	*Cypselurus oligolepis*	Exocoetidae	Beloniformes	Taiwan	Central Indo Pacific	Ho & Lin (2005)
	*Cypselurus* sp.	Exocoetidae	Beloniformes	Japan	Temperate Northern Pacific	Avdeev (1977)
*N. denticulatus* (Bassett-Smith, 1898) Vervoort, 1962	***Sphyraena jello***	Sphyraenidae	Order *incertae sedis* in Carangaria	**Trincomalee, Sri Lanka**	Western Indo Pacific	Bassett-Smith (1898)
	*Selar crumenophthalmus*	Carangidae	Carangiformes	Coral Sea; Port Moresby	Central Indo Pacific	Avdeev (1986)
	*Sphyraena chrysotaenia*	Sphyraenidae	Order *incertae sedis* in Carangaria	Mediterranean Sea, off Egypt	Temperate Northern Atlantic	El-Rashidy & Boxshall (2014)
	*Sphyraena jello*	Sphyraenidae	Order *incertae sedis* in Carangaria	Trivandrum, India	Western Indo Pacific	Pillai (1965)
	*Sphyraena jello*	Sphyraenidae	Order *incertae sedis* in Carangaria	Vietnam	Central Indo Pacific	Avdeev (1986)
*N. digitatus* Cressey & Collette, 1970	***Strongylura strongylura***	Belonidae	Beloniformes	**Penang, Malaysia**	Central Indo Pacific	Cressey & Collette (1970)
	*Strongylura leiura*	Belonidae	Beloniformes	Island of Java	Central Indo Pacific	Cressey & Collette (1970)
	*Strongylura leiura*	Belonidae	Beloniformes	Philippines	Central Indo Pacific	Cressey & Collette (1970)
	*Strongylura leiura*	Belonidae	Beloniformes	Gulf of Thailand	Central Indo Pacific	Cressey & Collette (1970)
	*Strongylura strongylura*	Belonidae	Beloniformes	Australia	Central Indo Pacific	Cressey & Collette (1970)
	*Strongylura strongylura*	Belonidae	Beloniformes	Calicut, Bombay	Western Indo Pacific	Cressey & Collette (1970)
	*Strongylura strongylura*	Belonidae	Beloniformes	Hong Kong	Central Indo Pacific	Cressey & Collette (1970)
	*Strongylura strongylura*	Belonidae	Beloniformes	India	Western Indo Pacific	Cressey & Collette (1970)
	*Tylosurus crocodilus*	Belonidae	Beloniformes	Northern Borneo	Central Indo Pacific	Cressey & Collette (1970)
	*Tylosurus punctulatus*	Belonidae	Beloniformes	New Guinea	Central Indo Pacific	Cressey & Collette (1970)
*N. elegans* Avdeev, 1977	***Scomberesox saurus***	Belonidae	Beloniformes	**Southeastern Pacific Ocean**	Eastern Indo Pacific	Avdeev (1977)
*N. epulus* Vervoort, 1962	***Plectorhinchus macrolepis***	Haemulidae	Lutjaniformes	**Niger Delta, Nigeria**	Tropical Atlantic	Vervoort (1962)
*N. exocoeti* Avdeev, 1978	***Exocoetus volitans***	Exocoetidae	Beloniformes	**Tropical Atlantic**	Tropical Atlantic	Avdeev (1978)
*N. fradei* Marques, 1965	***Sardinella maderensis***	Clupeidae	Clupeiformes	**Sao Tome, Gulf of Guinea**	Tropical Atlantic	Marques (1965)
	*Atherinomorus lacunosus* [as *Allanetta forskali* ]	Atherinidae	Atheriniformes	Arabian Gulf	Western Indo Pacific	Ho & Sey (1996)
	*Herklotsichthys punctatus*	Clupeidae	Clupeiformes	Mediterranean Sea, off Egypt	Temperate Northern Atlantic	El-Rashidy & Boxshall (2009)
	*Sardina pilchardus*	Clupeidae	Clupeiformes	Mediterranean Sea, off Egypt	Temperate Northern Atlantic	El-Rashidy & Boxshall (2009)
*N. gazzae* (Shen, 1957) Vervoort, 1969	***Gazza minuta***	Leiognathidae	Chaetodontiformes	**Hainan Island, China**	Central Indo Pacific	Shen (1957)
	*Siganus fuscescens*	Siganidae	Order *incertae sedis* in Eupercaria	Chiayi County, Taiwan	Central Indo Pacific	Lin & Ho (2008)
*N. gerresi* Pillai, 1973	***Gerres filamentosus***	Gerreidae	Gerreiformes	**Trivandrum, India**	Western Indo Pacific	Pillai (1973)
*N. gibber* (Shiino, 1957) Vervoort, 1962	***Tylosurus crocodilus*** **[as** ***Tylosus*** **[sic]** ***giganteus*]**	Belonidae	Beloniformes	**Owase, Mie, Japan**	Temperate Northern Pacific	Shiino (1957)
	*Ablennes hians*	Belonidae	Beloniformes	Andaman Island	Western Indo Pacific	Cressey & Collette (1970)
	*Ablennes hians*	Belonidae	Beloniformes	Bay of Bengal	Western Indo Pacific	Cressey & Collette (1970)
	*Ablennes hians*	Belonidae	Beloniformes	Borneo	Central Indo Pacific	Cressey & Collette (1970)
	*Ablennes hians*	Belonidae	Beloniformes	Japan	Temperate Northern Pacific	Cressey & Collette (1970)
	*Ablennes hians*	Belonidae	Beloniformes	Philippines	Central Indo Pacific	Cressey & Collette (1970)
	*Ablennes hians*	Belonidae	Beloniformes	Torres Straits	Central Indo Pacific	Cressey & Collette (1970)
	*Belone belone*	Belonidae	Beloniformes	Funchal, Madeira	Temperate Northern Atlantic	Cressey & Collette (1970)
	*Belone svetovidovi*	Belonidae	Beloniformes	Genoa, Italy	Temperate Northern Atlantic	Cressey & Collette (1970)
	*Belone svetovidovi*	Belonidae	Beloniformes	Tunisia	Temperate Northern Atlantic	Cressey & Collette (1970)
	*Belones platyura*	Belonidae	Beloniformes	Eniwetok Atoll	Eastern Indo Pacific	Lewis (1968)
	*Euleptorhamphus viridis*	Hemiramphidae	Beloniformes	Timor Sea	Central Indo Pacific	Avdeev (1977)
	*Platybelone argalus*	Belonidae	Beloniformes	Aldabra	Western Indo Pacific	Cressey & Collette (1970)
	*Platybelone argalus*	Belonidae	Beloniformes	Ascension Island	Tropical Atlantic	Cressey & Collette (1970)
	*Platybelone argalus*	Belonidae	Beloniformes	Fakaofo Atoll	Eastern Indo Pacific	Cressey & Collette (1970)
	*Platybelone argalus*	Belonidae	Beloniformes	Fanning Island	Eastern Indo Pacific	Cressey & Collette (1970)
	*Platybelone argalus*	Belonidae	Beloniformes	Gulf of Guinea	Tropical Atlantic	Cressey & Collette (1970)
	*Platybelone argalus*	Belonidae	Beloniformes	Line Islands	Eastern Indo Pacific	Cressey & Collette (1970)
	*Platybelone argalus*	Belonidae	Beloniformes	Marshall Island	Eastern Indo Pacific	Cressey & Collette (1970)
	*Platybelone argalus*	Belonidae	Beloniformes	Somoa	Eastern Indo Pacific	Cressey & Collette (1970)
	*Platybelone argalus*	Belonidae	Beloniformes	Tokelau Island	Eastern Indo Pacific	Cressey & Collette (1970)
	*Strongylura leiura*	Belonidae	Beloniformes	Taiwan	Central Indo Pacific	Ho & Lin (2005)
	*Tylosurus acus*	Belonidae	Beloniformes	Taiwan	Central Indo Pacific	Cressey & Collette (1970)
	*Tylosurus crocodilus*	Belonidae	Beloniformes	Kerala, India	Central Indo Pacific	Cressey & Collette (1970)
	*Tylosurus crocodilus*	Belonidae	Beloniformes	Madagascar	Western Indo Pacific	Cressey & Collette (1970)
	*Tylosurus crocodilus*	Belonidae	Beloniformes	Red Sea	Western Indo Pacific	Cressey & Collette (1970)
	*Tylosurus crocodilus*	Belonidae	Beloniformes	Seychelles	Western Indo Pacific	Cressey & Collette (1970)
	*Tylosurus crocodilus*	Belonidae	Beloniformes	Zanzibar	Western Indo Pacific	Cressey & Collette (1970)
*N. ilhoikimi* Venmathi Maran et al., 2014	***Tenualosa ilisha***	Clupeidae	Clupeiformes	**Al-Faw City, Iraq**	Western Indo Pacific	Venmathi Maran et al. (2014)
*N. johndaveorum* n. sp.	***Gerres subfasciatus***	Gerreidae	Gerreiformes	**Moreton Bay, Queensland, Australia 27°22′S, 153°13′E**	Central Indo Pacific	Present study
	*Gerres oyena*	Gerreidae	Gerreiformes	Moreton Bay, Queensland, Australia 27°26′S, 153°24′E	Central Indo Pacific	Present study
*N. kanagurta* (Pillai, 1965) Cressey & Cressey, 1980	***Rastrelliger kanagurta***	Scombridae	Scombriformes	**Kerala, India**	Central Indo Pacific	Pillai (1965)
	*Rastrelliger faughni*	Scombridae	Scombriformes	Philippines	Central Indo Pacific	Cressey & Cressey (1980)
	*Rastrelliger kanagurta*	Scombridae	Scombriformes	China	Central Indo Pacific	Cressey & Cressey (1980)
	*Rastrelliger kanagurta*	Scombridae	Scombriformes	Madras, India	Western Indo Pacific	Cressey & Cressey (1980)
	*Rastrelliger kanagurta*	Scombridae	Scombriformes	Red Sea	Western Indo Pacific	Cressey & Cressey (1980)
	*Rastrelliger kanagurta*	Scombridae	Scombriformes	Taiwan	Central Indo Pacific	Ho & Lin (2005)
	*Scomber japonicus*	Scombridae	Scombriformes	Gulf of Mannar, India	Western Indo Pacific	Avdeev (1978)
*N. lateolabracis* (Yamaguti & Yamasu, 1959) Vervoort, 1962	***Lateolabrax japonicus***	Sillaginidae	Order *incertae sedis* in Eupercaria	**Inland Sea, Japan**	Temperate Northern Pacific	Yamaguti & Yamasu (1959)
	*Lateolabrax japonicus*	Sillaginidae	Order *incertae sedis* in Eupercaria	Ashai River, Japan	Temperate Northern Pacific	Ho, Do & Kasahara (1983)
	*Sillago sihama*	Sillaginidae	Order *incertae sedis* in Eupercaria	Taiwan	Central Indo Pacific	Ho & Lin (2005)
*N. leiognathicola* El-Rashidy & Boxshall, 2014	***Leiognathus klunzingeri***	Leiognathidae	Chaetodontiformes	**Mediterranean Sea, off Egypt**	Temperate Northern Atlantic	El-Rashidy & Boxshall (2014)
*N. lizae* Ho & Lin, 2005	***Liza macrolepis***	Mugilidae	Mugiliformes	**Taiwan**	Central Indo Pacific	Ho & Lin (2005)
*N. longisaccus* Ho & Lin, 2005	***Thryssa hamiltonii***	Engraulidae	Clupeiformes	**Taiwan**	Central Indo Pacific	Ho & Lin (2005)
*N. marginatus* Avdeev, 1986	unknown	NA	NA	10°07′S 145°57′E	Central Indo Pacific	Avdeev (1986)
*N. monodi* El-Rashidy & Boxshall, 2014	***Hemiramphus far***	Hemiramphidae	Beloniformes	**Madagascar**	Western Indo Pacific	Monod (1970)
*N. multispinosus* (Gnanamuthu, 1949) Vervoort, 1962	***Dussumiera acuta***	Dussumieridae	Clupeiformes	**Madras, India**	Western Indo Pacific	Gnanamuthu (1949)
	*Dussumiera elopsoides*	Dussumieridae	Clupeiformes	Kerala, India	Western Indo Pacific	Pillai (1985)
*N. neomediterraneus* El-Rashidy & Boxshall, 2011	***Siganus rivulatus***	Siganidae	Order *incertae sedis* in Eupercaria	**Mediterranean Sea, off Egypt**	Temperate Northern Atlantic	El-Rashidy & Boxshall (2011)
*N. ovalis* Avdeev, 1977	***Siganus stellatus***	Siganidae	Order *incertae sedis* in Eupercaria	**Mannar Straits, Indian Ocean**	Western Indo Pacific	Avdeev (1977)
*N. oxyporhamphi* Avdeev, 1977	***Oxyporhamphus micropterus***	Hemiramphidae	Beloniformes	**Galapagos Islands**	Tropical Eastern Pacific	Avdeev (1977)
*N. paruchini* Avdeev, 1978	***Exocoetus volitans***	Exocoetidae	Beloniformes	**Tropical Atlantic**	Tropical Atlantic	Avdeev (1978)
*N. pulicatensis* Kaliyamurthy, 1990	***Hyporhamphus quoyi***	Hemiramphidae	Beloniformes	**Pulicat Lake, India**	Western Indo Pacific	Kaliyamurthy (1990)
*N. quadriceros* Pillai, 1973	***Gazza minuta***	Leiognathidae	Chaetodontiformes	**Trivandrum, India**	Western Indo Pacific	Pillai (1973)
*N. saetiger* (Wilson, 1911) Vervoort, 1962	***Exocoetus volitans***	Exocoetidae	Beloniformes	**Massachusetts, USA**	Temperate Northern Atlantic	Wilson (1911)
	*Menidia menidia*	Atherinopsidae	Atheriniformes	North Carolina, USA	Temperate Northern Atlantic	Pearse (1947)
*N. sagaxi* Avdeev, 1986	***Sardinops sagax***	Clupeidae	Clupeiformes	**South Kuril Island 43°42′N 148°30′E**	Temperate Northern Pacific	Avdeev (1986)
*N. scomberesocis* (Krøyer, 1863) Vervoort, 1962	***Scomberesox*sp.**	Belonidae	Beloniformes	**Atlantic Ocean**	NA	Krøyer (1863)
	*Scomberesox saurus [as S. rondeletii]*	Belonidae	Beloniformes	Cabo Creus, Spain	Temperate Northern Atlantic	Deboutteville & Nunes-Ruivo (1958)
*N. sigani* Hameed & Kumar, 1988	***Siganus canaliculatus [as Siganus oramin]***	Siganidae	Order *incertae sedis* in Eupercaria	**Trivandrum, India**	Western Indo Pacific	Hameed & Kumar (1988)
*N. teres* (Wilson, 1911) Pillai, 1967	***Brevoortia tyrannus***	Clupeidae	Clupeiformes	**Massachusetts, USA**	Temperate Northern Atlantic	Wilson (1911)
	*Brevoortia smithi*	Clupeidae	Clupeiformes	Charlotte Harbor, Florida, USA	Tropical Atlantic	Cressey (1983)
	*Brevoortia tyrannus*	Clupeidae	Clupeiformes	Charlotte Harbor, Florida, USA	Tropical Atlantic	Cressey (1983)
*N. thambus* Ho, Do & Kasahara, 1983	***Konosirus punctatus***	Clupeidae	Clupeiformes	**Ashai River, Japan**	Temperate Northern Pacific	Ho, Do & Kasahara (1983)
*N. triceros* (Bassett-Smith, 1898) Vervoort, 1962	***Pampus argenteus [as Stromateus cinereus]***	Stromateidae	Scombriformes	**Arabian Sea**	Western Indo Pacific	Bassett-Smith (1898)
	*Lobotes surinamensis*	Lobotidae	Lobotiformes	Taiwan	Central Indo Pacific	Ho & Lin (2005)
	*Pampus argenteus*	Stromateidae	Scombriformes	Ashai River, Japan	Temperate Northern Pacific	Ho, Do & Kasahara (1983)
	*Pampus argenteus*	Stromateidae	Scombriformes	Kuwait Bay	Western Indo Pacific	Ho, Kim & Sey (2000)
	*Pampus argenteus*	Stromateidae	Scombriformes	Pacific Ocean	NA	Yamaguti (1939)
	*Pampus argenteus*	Stromateidae	Scombriformes	Persian Gulf, Iran	Western Indo Pacific	Khosheghbal & Pazooki (2015)
	*Pampus argenteus*	Stromateidae	Scombriformes	Taiwan	Central Indo Pacific	Ho & Lin (2005)
	*Pampus argenteus [as Stromateoides argentus]*	Stromateidae	Scombriformes	Kwangtung, China	Central Indo Pacific	Shen (1957)
*N. trichiuri* Pillai & Natarajan, 1977	***Lepturacanthus savala [as Trichiurus savala]***	Trichiuridae	Scombriformes	**Trivandrum, India**	Western Indo Pacific	Pillai & Natarajan (1977)
	*Lepturacanthus savala [as Trichiurus savala]*	Trichiuridae	Scombriformes	Trivandrum, India	Western Indo Pacific	Hameed & Kumar (1988)
	*Trichiurus lepturus*	Trichiuridae	Scombriformes	Taiwan	Central Indo Pacific	Ho & Lin (2005)
*N. vervoorti* Avdeev, 1986	***Ilisha elongata***	Pristigasteridae	Clupeiformes	**Vietnam**	Central Indo Pacific	Avdeev (1986)

### *Nothobomolochus* host associations and biogeography

The hosts and localities of all known species of *Nothobomolochus* are summarized in Table 2. The 39 species of *Nothobomolochus* have collectively been reported 112 times, excluding the suspect host reports identified by El-Rashidy & Boxshall (2014). Following the revised classification of bony fishes by Betancur et al. (2017), the genus *Nothobomolochus* parasitizes at least 11 orders of fish (Acanthuriformes, Atheriniformes, Beloniformes, Carangiformes, Chaetodontiformes, Clupeiformes, Gerreiformes, Lobotiformes, Lutjaniformes, Mugiliformes, and Scombriformes) and 22 different families, with some host orders currently *incertae sedis* in the Eupercaria and Carangaria series. The vast majority of host reports come from the orders Beloniformes (*n* = 56; 50%), Scombriformes (*n* = 17; 15%), and Clupeiformes (*n* = 14; 13%), with 6 or fewer reports (<5%) from the other 8 host orders. Most reports are from the families Belonidae (*n* = 41; 37%), followed by the Exocoetidae (*n* = 11; 10%), Clupeidae (*n* = 9; 8%), and Scombridae and Stromateidae (*n* = 7 each; 6%) with 4 or fewer reports (<4%) from the 17 other host families.

Following the marine realms established by Spalding et al. (2007), we observe the following distribution for the genus *Nothobomolochus* as currently understood. Of the 112 reports of species of *Nothobomolochus,* 36 (33%) come from the Central Indo-Pacific realm, followed by 30 (27%) from the Western Indo-Pacific, 13 (12%) in the Temperate Northern Atlantic, and 10 or fewer (<9% each) in the following 5 realms: Temperate Northern Pacific (10), Tropical Atlantic (10), Eastern Indo-Pacific (9), Temperate South American (1), and Tropical Eastern Pacific (1). The report of *N. scomberesocis* from the Atlantic Ocean by Krøyer (1863) and *N. triceros* from the Pacific Ocean by Yamaguti (1939) could not be unambiguously assigned to a realm because the reports lack precise locality information. There are currently no reports of *Nothobomolochus* from the Temperate Australasia, Temperate Southern Africa, Arctic, or Southern Ocean realms.

**Table:** 

**Figure 5:**
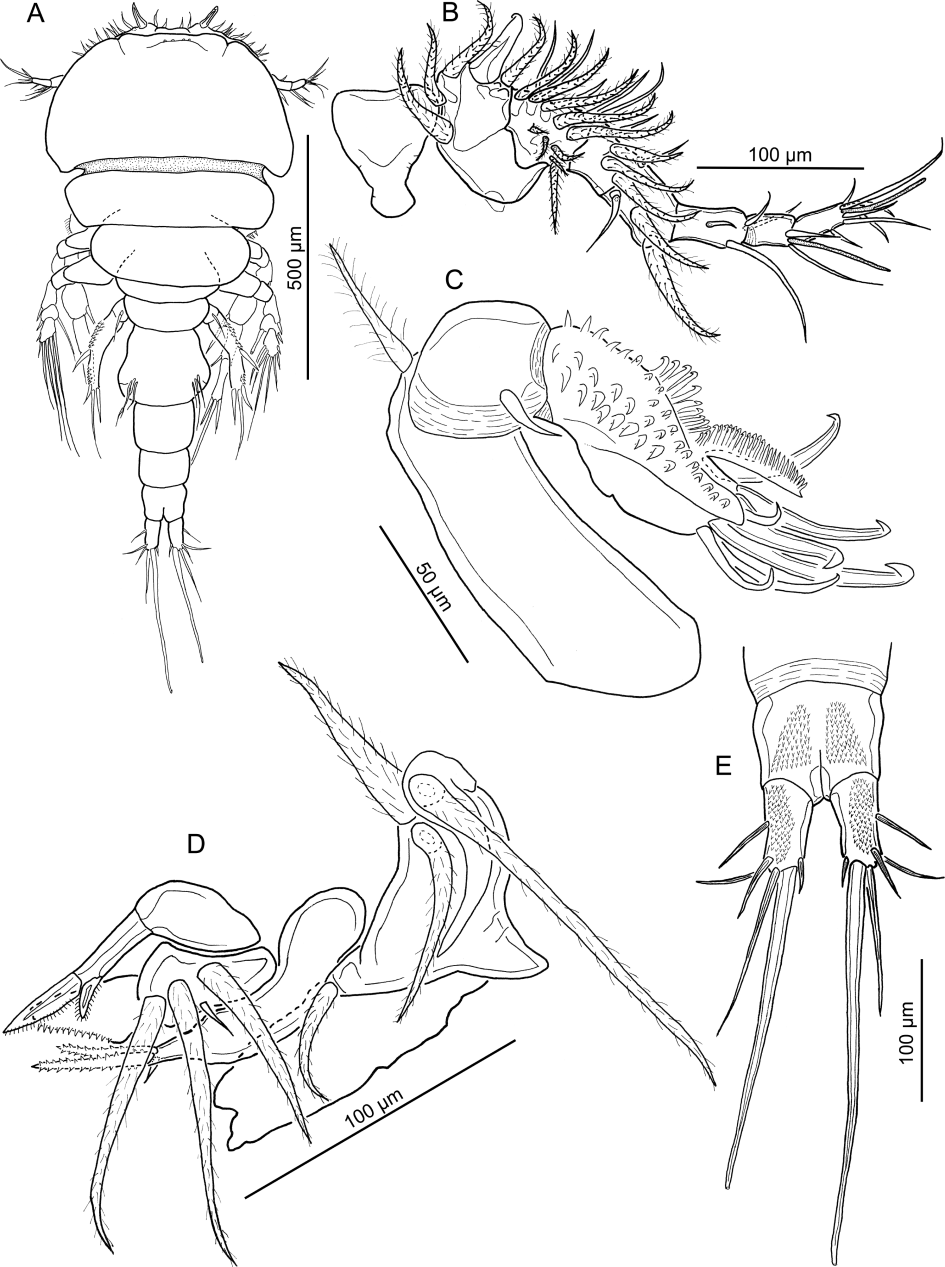
Line drawings of *Unicolax longicrus* n. sp. female. (A) Habitus, dorsal view (holotype QM W29434). (B) Antennule, ventral view (holotype QM W29434). (C) Antenna, ventral view (paratype NHMUK 2018.198). (D) Oral area showing mandible, maxillule, paragnath, maxilla, and maxilliped, *in situ* (holotype QM W29434). (E) Caudal rami and anal somite, ventral view (holotype QM W29434). Drawing credit: James P. Bernot.

**Figure 6:**
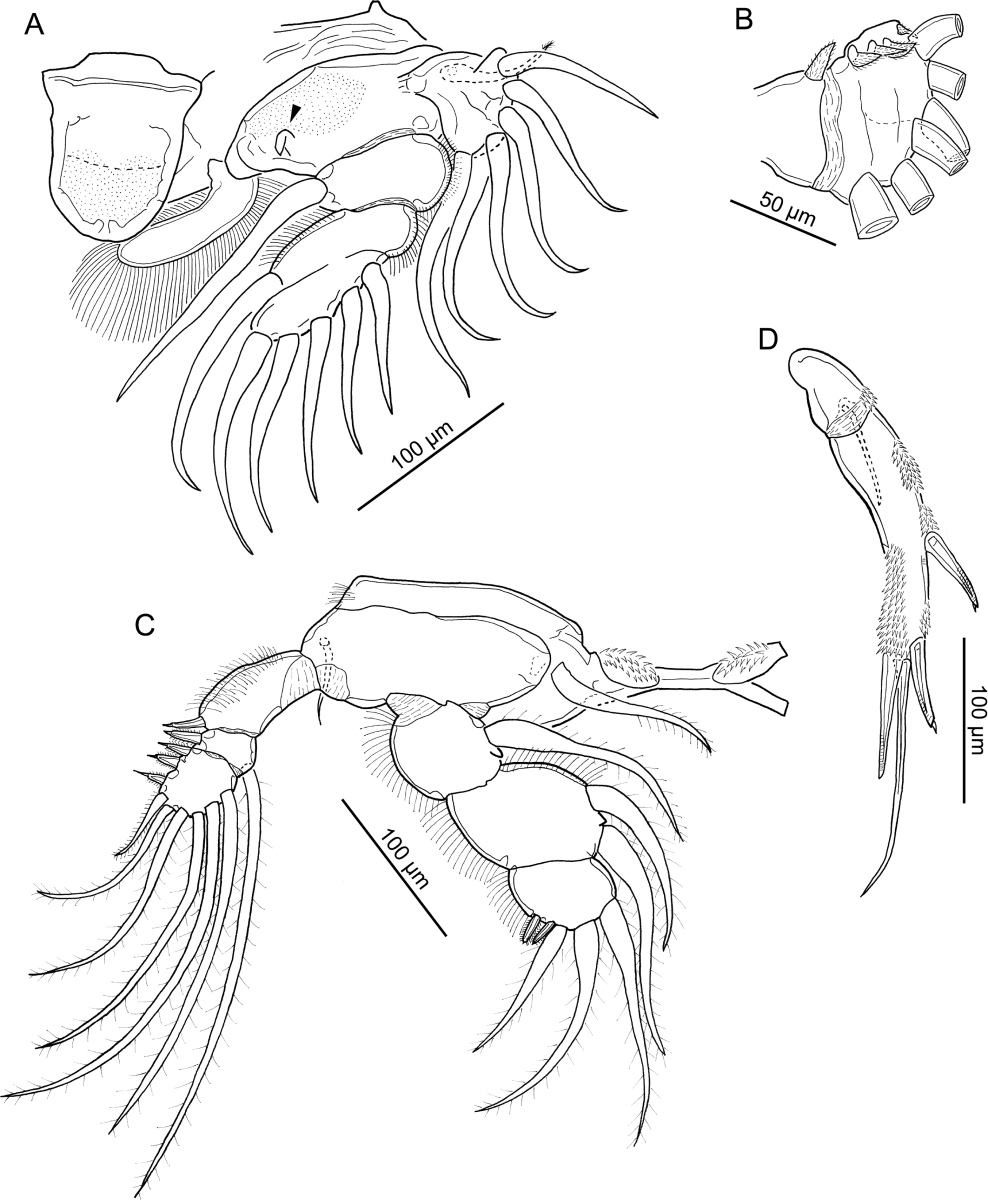
Line drawings of *Unicolax longicrus* n. sp. female. (A) Leg 1 and intercoxal sclerite, ventral view (holotype QM W29434); plumosity on setae not illustrated; arrowhead indicating inner seta represented by hooked tubercle. (B) Outer spines on Leg 1 exopod, dorsal view (paratype NHMUK 2018.198). (C) Leg 2 and intercoxal sclerite, ventral view (paratype NHMUK 2018.198). (D) Leg 5, ventral view (holotype QM W29434). Drawing credit: James P. Bernot.

**Figure 7:**
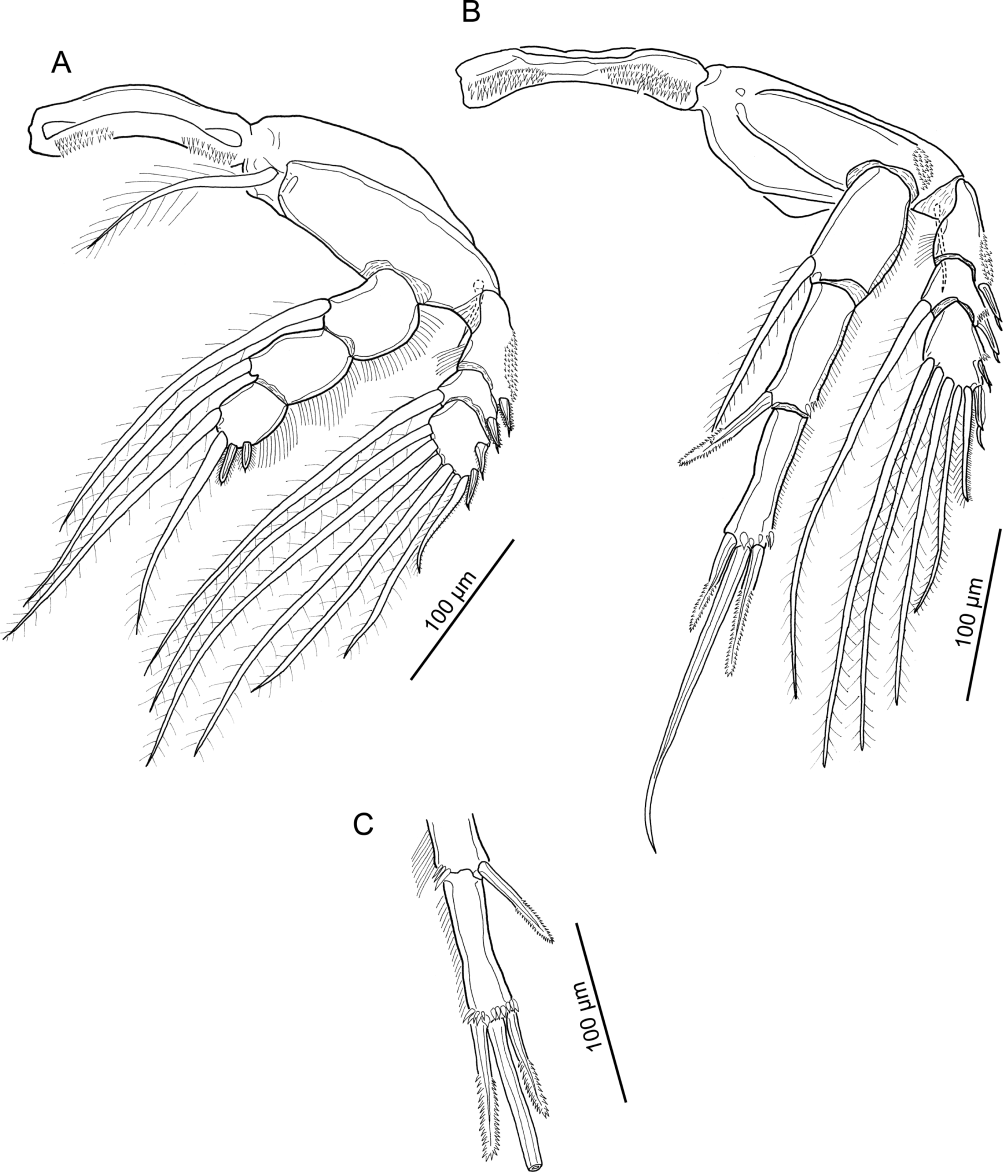
Line drawings of *Unicolax longicrus* n. sp. female. (A) Leg 3 and intercoxal sclerite, ventral view (holotype QM W29434). (B) Leg 4 and intercoxal sclerite, ventral view (holotype QM W29434). (C) Detail of leg 4 endopod distal armature, same specimen as 7B, opposite leg (holotype QM W29434).

**Figure 8:**
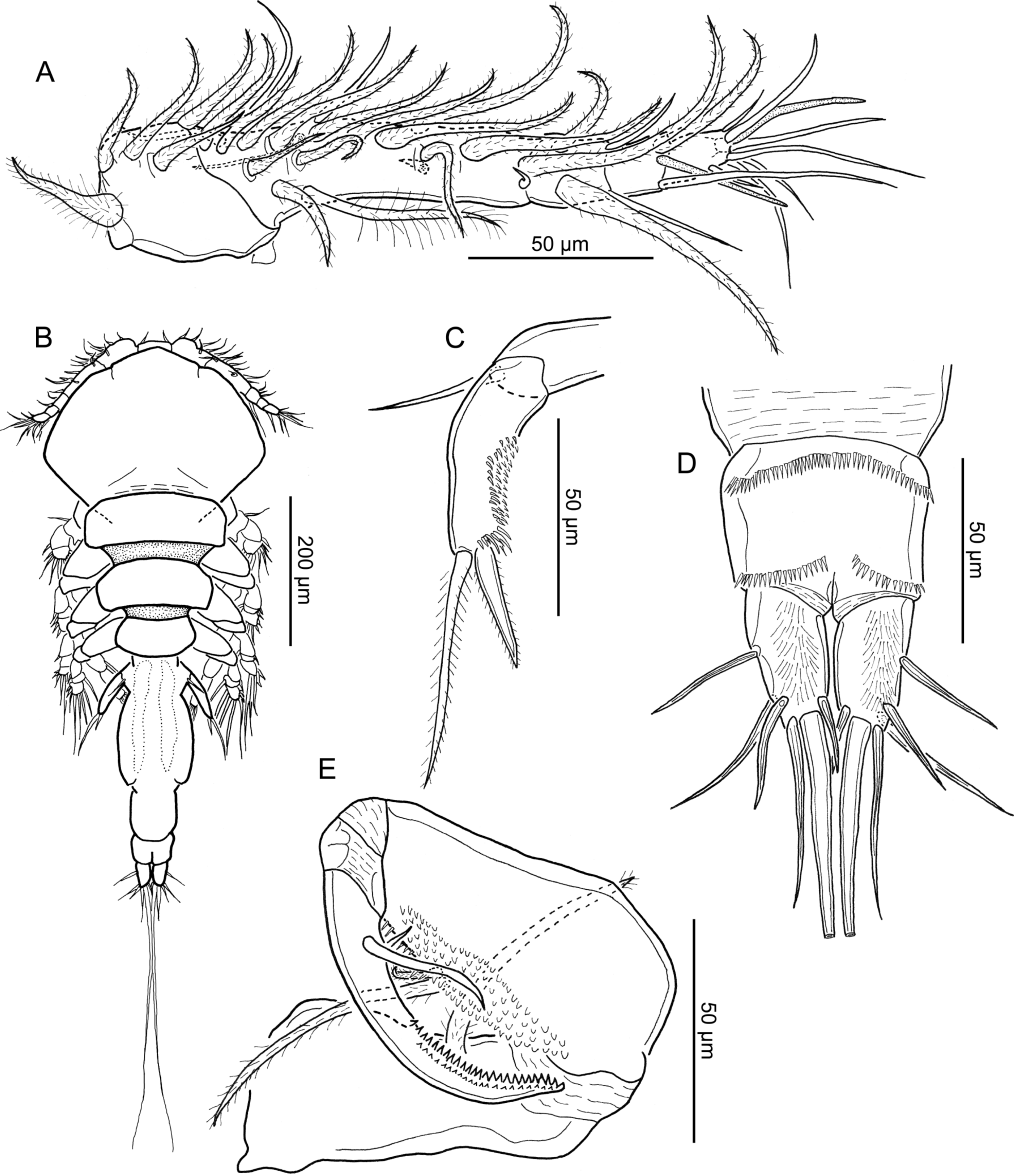
Line drawings of *Unicolax longicrus* n. sp. male. (A) Antennule, ventral view (allotype QM W29436). (B) Habitus, dorsal view (allotype QM W29436). (C) Leg 5, ventral view (allotype QM W29436). (D) Caudal rami and anal somite, ventral view (allotype QM W29436). (E) Maxilliped, ventral view (allotype QM W29436). Drawing credit: James P. Bernot.

**Figure 9:**
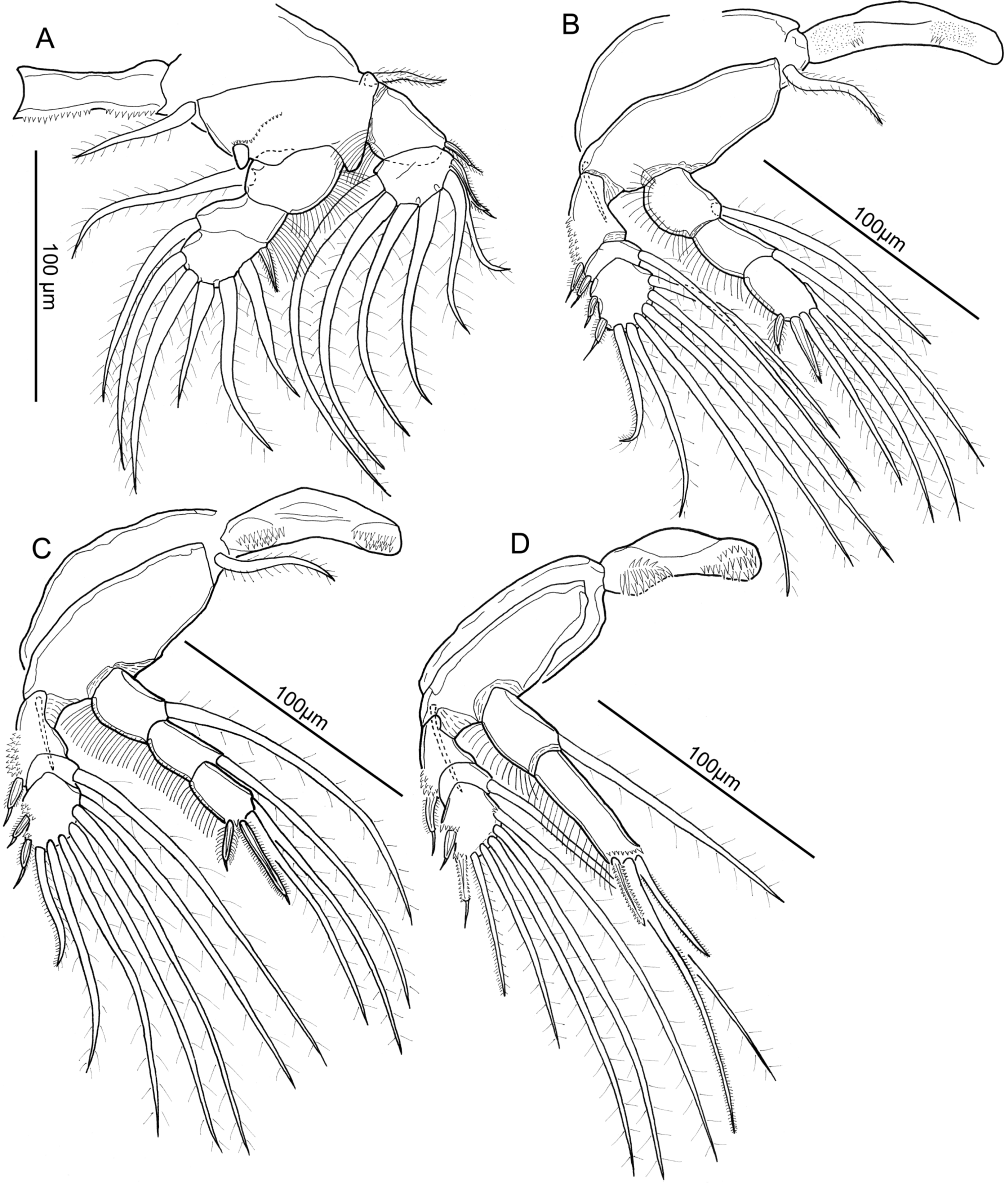
Line drawings of *Unicolax longicrus* n. sp. male. (A) Leg 1 and intercoxal sclerite, ventral view (allotype QM W29436). (B) Leg 2 and intercoxal sclerite, ventral view (allotype QM W29436). (C) Leg 3 and intercoxal sclerite, ventral view (allotype QM W29436). (D) Leg 4 and intercoxal sclerite, ventral view (allotype QM W29436). Drawing credit: James P. Bernot.

Genus *Unicolax*Cressey & Cressey, 1980
*Unicolax longicrus* n. sp.
(Figs. 5–9).

*LSID:* urn:lsid:zoobank.org:act:0E24C5F0-29C1-49C9-830D-7124D27D3FAE

*Type-host*: *Sillago maculata* Quoy & Gaimard, 1824 (Order-level *incertae sedis* in Eupercaria: Sillaginidae).

*Other host: Sillago ciliata* Cuvier, 1829 (Order-level *incertae sedis* in Eupercaria: Sillaginidae).

*Type-locality*: Moreton Bay, Queensland, Australia (27°26′S, 153°24′E).

*Site*: Nasal cavity.

*Type-material*: Holotype female (QM W29434) and 5 female paratypes (1 paratype QM W29435; 2 paratypes NHMUK 2018.198–2018.199; 2 paratypes USNM 1532294–1532295). Allotype male (QM W29436) and 4 male paratypes (2 paratypes NHMUK 2018.200–2018.201; 2 paratypes USNM 1532296–1532297).

*Etymology*: The name of this species is derived from the Latin *longus* (long) and *crus* (leg), in reference to the elongate endopodal segments of leg 4.

Adult female.

Body (Fig. 5A) 980–1430 (1171 ± 183; *n* = 6) long, measured along midline from frontal margin of rostrum to posterior margin of caudal rami excluding caudal setae; greatest width 432–600 (501 ± 64; 6) at posterior of dorsal cephalothoracic shield. Prosome 490–770 (641 ± 115; 6) long by 432–600 (501 ± 64; 6) wide, comprising broad cephalothorax and 3 free pedigerous somites. Urosome 450–650 (553 ± 85; 6) long by 162–210 (192 ± 19; 6) wide, comprising 5th pedigerous somite, genital double-somite, and 3 free abdominal somites. Genital double-somite (Fig. 5A) bearing paired genital apertures dorso-laterally. Anal somite (Fig. 5E) bearing paired caudal rami; ornamented with 2 patches of spinules, anal slit deeply incised. Caudal rami (Fig. 5E) longer than wide, bearing principal seta and 5 smaller setae, ornamented with patch of spinules. Egg sacs elongate, multiseriate. Swimming leg armature summarized in Table 3.

Antennule (Fig. 5B) 7-segmented; first segment heavily sclerotized at base; second to fourth segments partially fused, 3 distal segments cylindrical. First segment bearing single broad, spine-like fourth seta plus 4 hirsute setae. Second segment bearing 5 hirsute setae and 5 naked setae along anterior margin, and 5 hirsute setae arrayed across ventral surface extending posteriorly. Third segment bearing 3 hirsute and 2 naked setae. Fourth segment bearing 2 hirsute plus 1 naked seta. Distal 3 segments with setal formula: 4; 2 + 1 ae; 7 + 1 ae.

**Table 3:** *Unicolax longicrus* n. sp. swimming leg armature.

	Coxa	Basis	Exopod	Endopod
Female				
Leg 1	0-1	1-I	I-0; IV, 6	0-1; 0-1; 5
Leg 2	0-1	1-0	I-0; I-1; III, 1, 5	0-1; 0-2; II, 3
Leg 3	0-1	1-0	I-0; I-1; II, 1, 5	0-1; 0-1; II, 2
Leg 4	0-0	1-0	I-0; I-1; II, 1, 4	0-1; 0-I; III
Male				
Leg 1	0-1	1-I	I-0; I, 1, 5	0-1; 0-1; I, 5
Leg 2	0-1	1-0	I-0; I-1; II, 1, 5	0-1; 0-1; II, 3
Leg 3	0-1	1-0	I-0; 0-1; II, 1, 5	0-1; 0-1; II, 2
Leg 4	0-0	1-0	I-0; 0-1; II, 1, 4	0-1; III

Antenna (Fig. 5C) uniramous, 3-segmented, comprising elongate coxobasis bearing hirsute seta, short first endopodal segment bearing naked seta, and heavily armed compound distal segment. Distal segment ornamented with 3 irregular rows of spinules, 2 rows of spinules extending onto elongate distal process, bearing one pectinate process extending distally and enlarged teeth medially; distal armature comprising 4 claw-like setae, 2 elongate naked setae, and 1 short naked seta.

Mandible (Fig. 5D) bearing two spinulate blades of unequal length. Paragnath (Fig. 5D) tapering distally, with tooth-like spinules on posterior margin. Maxillule (Fig. 5D) consisting of irregular lobe with 3 large hirsute setae and 1 small naked seta. Maxilla (Fig. 5D) indistinctly 2-segmented; proximal segment unarmed; distal segment with 2 unequal spinulate apical processes and 1 small naked seta dorsally. Maxilliped (Fig. 5D) 3-segmented, comprising long syncoxa armed with hirsute seta; middle segment tapering distally, armed with 2 large hirsute setae on medial margin; distal segment incorporated into claw and bearing long hirsute seta; claw simple, lacking accessory process.

Leg 1 (Fig. 6A) biramous, with flattened lamellate rami; members of leg pair joined by intercoxal sclerite bearing lingulate process ornamented with patch of small spinules distally. Coxa with numerous ridge-like cuticular thickenings and inflated inner plumose seta. Basis ornamented with patch of small spinules proximally, outer plumose seta, and inner seta reduced to hooked tubercle (arrowhead in Fig. 6A). Exopod (Figs. 6A and 6B) indistinctly 2-segmented; proximal segment armed with short hirsute spine on dorsal surface; compound distal segment formed by fusion of segments 2 and 3, bearing 6 plumose setae (plumosity not figured in Figs. 6A and 6B), and 4 short hirsute spines on dorsal surface (Fig. 6B). Endopod 3-segmented; all segments flattened and expanded transversely; first segment with inner plumose seta and hair-like setules on outer margin; second segment partially fused to third, with inner plumose seta and hair-like setules on inner and outer margins; third segment armed with 5 plumose setae.

Leg 2 (Fig. 6C) biramous; intercoxal sclerite ornamented with paired lateral fields of spinules on raised expansions. Coxa with inner plumose seta and patch of hair-like setules on outer margin. Basis with outer naked seta. Exopod 3-segmented; first segment bearing outer spine and ornamented with hair-like setules in patch on outer surface; distal segments with setal formula I-1; III, 1, 5. All spines bearing subterminal flagellum, and all but segment 1 spine with bilateral spinulation. Endopod 3-segmented; first segment with plumose inner seta and hair-like setules on outer margin; second segment with 2 plumose inner setae and hair-like setules on inner and outer margins; third segment with hair-like setules on outer margin, 3 plumose setae, and 2 spines with very fine spinulation on margins.

Leg 3 (Fig. 7A) biramous; intercoxal sclerite with rows of spinules in paired lateral fields and cuticular folds. Coxa with inner plumose seta. Basis with outer naked seta. Exopod 3-segmented; first segment with outer spine, segment ornamented with patch of spinules on outer margin and hair-like setules on inner margin; second segment with outer spine and inner seta; third segment with formula: II, 1, 5. All outer spines with bilateral spinulation and subterminal flagellum; proximal 2 spines with more robust bilateral serrations. Endopod 3-segmented; first and second segments each with plumose inner seta and hair-like setules along outer margin; third segment with 2 inner setae plus 2 spines bearing fine spinulation bilaterally, and ornamented with hair-like setules along outer margin.

Leg 4 (Fig. 7B) biramous, with intercoxal sclerite ornamented with spinules in paired lateral fields. Coxa lacking inner seta. Basis with naked outer seta and ornamented with patch of spinules near base of exopod. Exopod 3-segmented; first segment with outer spine bearing subterminal flagellum, segment ornamented with patch of spinules on outer margin and hair-like setules along inner margin; second segment bearing plumose seta, outer spine with flagellum, and spinules at base of spine; third segment with setal formula: II, 1, 4; all spines with flagellum; spinules present at base of proximal spine; distal spine bearing flange on outer margin. Endopod 3-segmented; all segments ornamented with row of short hair-like setules along outer margin; first segment bearing plumose seta extending to 30% of length of third segment; second segment (Figs. 7B and 7C) elongate, length:width ratio 2.5, bearing large spinules in cluster on outer distal margin and spine-like seta extending just beyond midline of distal segment; seta with marginal serrations on distal half; third segment (Figs. 7B and 7C) highly elongate, length:width ratio 3.8, bearing 3 spines along distal margin with large spinules at bases of spines; inner and outer distal spines of unequal length, outer spine as long as segment and 20% longer than inner; both ornamented with lateral serrations distally; middle spine naked, more than 2x longer than outer distal spine.

Leg 5 (Fig. 6D) 2-segmented: protopodal segment with naked outer seta and small patch of spinules on outer distal margin; exopodal segment with 4 patches of spinules and 4 setal elements; outer 2 spines each with subterminal flagellum; terminal seta and inner spine naked; inner spine approximately 30% longer than outer; terminal seta markedly longer than other elements, just longer than segment, nearly twice as long as inner spine and 2.5x longer than outer spine. Leg 6 (Fig. 5A) represented by 3 setae near oviduct opening.

Adult male.

Body (Fig. 8B) 650–770 (715 ± 46; *n* = 5) long, measured along midline from frontal margin of rostrum to posterior margin of caudal rami excluding caudal setae; greatest width 270–320 (291 ± 22; 5) at posterior of dorsal cephalothoracic shield. Prosome 370–460 (410 ± 34; 5) long by 270–320 (291 ± 22; 5) wide, comprising broad cephalothorax and 3 free pedigerous somites. Urosome 270–344 (306 ± 30; 5) long by 80–130 (108 ± 22; 5) wide, comprising fused fifth pedigerous somite, genital somite, and 2 abdominal somites, plus 1 free abdominal (anal) somite. Anal somite (Fig. 8D) bearing paired caudal rami; ornamented with anterior row of spinules plus 2 lateral rows of spinules posteriorly. Caudal rami longer than wide, bearing principal seta and 5 smaller setae, ventral surface ornamented with extensive patch of hair-like setules. Swimming leg armature summarized in Table 3.

Most appendages sexually dimorphic, except antenna, mandible, paragnath, maxillule, and maxilla as in female. Antennule (Fig. 8A) 5-segmented; first segment heavily sclerotized at base; second to fourth segments partially fused, 3 distal segments cylindrical. First segment bearing 5 hirsute setae. Second segment bearing 13 hirsute setae and 2 naked setae along anterior margin, and 2 hirsute setae and 1 naked seta on posterior ventral surface; 2 long and 1 short seta present on dorsal surface. Third segment with 2 hirsute and 2 naked setae. Fourth segment bearing 1 hirsute seta, 1 naked seta, and 1 aesthetasc. Distal segment with setal formula: 7 + 1 ae; setae naked.

Maxilliped (Fig. 8E) 3-segmented, comprising long syncoxa bearing hirsute seta; middle segment tapering distally, with 1 long and 1 short hirsute setae on medial margin, ornamented with patch of blunt spinules along inner surface and row of elongate spinules along inner margin distally; distal segment incorporated into claw and bearing 1 long and 1 short naked setae; claw with 2 rows of teeth in distal half of concave margin.

Leg 1 (Fig. 9A) biramous, with flattened lamellate rami; intercoxal sclerite flattened, ornamented with row of spinules along free posterior margin. Coxa with inner plumose seta. Basis ornamented with row of spinules proximally, outer hirsute seta, and short inner seta reduced to rounded tubercle. Exopod 2-segmented; proximal segment with outer spine and hair-like setules along inner margin; spine armed with subterminal flagellum and bilateral spinulation; distal segment bearing 6 plumose setae and 1 spine with subterminal flagellum and bilateral spinulation. Endopod indistinctly 3-segmented; first segment with inner plumose seta and hair-like setules on outer margin; second segment partially fused to third, with inner plumose seta and hair-like setules on outer margins; third segment armed with 5 plumose setae and 1 spine with bilateral spinulation; terminal seta shorter than adjacent 4 setae.

Leg 2 (Fig. 9B) biramous; intercoxal sclerite ornamented with paired lateral fields of small spinules with three larger spinules on inner margin of each field. Coxa with inner plumose seta. Basis with outer seta. Exopod 3-segmented; first segment bearing outer spine and spinules in patch on outer margin; distal segments with setal formula I-1; II, 1, 5; all outer spines bearing subterminal flagellum and bilateral spinulation. Endopod 3-segmented; first and second segments each with plumose inner seta and hair-like setules on outer margin; third segment with hair-like setules on outer margin and setal formula II, 3; spines with subterminal flagellum and bilateral spinulation; terminal spine twice as long as proximal, with spinulation only on distal 25%.

Leg 3 (Fig. 9C) biramous; intercoxal sclerite ornamented with cuticular folds and paired lateral fields of spinules on raised expansions. Coxa with plumose inner seta. Basis with outer seta. Exopod 3-segmented; first segment with outer spine and patch of spinules on outer margin; second segment lacking outer spine, armed with inner seta; third segment with formula: II, 1, 5, ornamented with small patches of spinules at base of spines; all spines with bilateral spinulation and subterminal flagellum. Endopod 3-segmented; first and second segments each with plumose inner seta and hair-like setules along outer margin; third segment ornamented with hair-like setules along outer margin and 2 inner setae plus 2 spines bearing subterminal flagella and bilateral spinulation; terminal spine twice as long as proximal spine.

Leg 4 (Fig. 9D) biramous, with intercoxal sclerite ornamented with spinules in paired lateral fields on raised bases. Coxa lacking inner seta. Basis with outer seta. Exopod 3-segmented; first segment with outer spine bearing subterminal flagellum and bilateral spinulation, segment ornamented with patch of spinules on outer margin; second segment lacking outer spine but bearing plumose seta; third segment with setal formula: II, 1, 4; small patches of spinules present at bases of spines and innermost seta; all spines with subterminal flagella; first spine otherwise unornamented; terminal spine with serrated margins; terminal setal element with asymmetrical spinulation on inner and outer margins. Endopod 2-segmented; both segments ornamented with row of long hair-like setules along outer margin; first segment bearing plumose seta; second segment elongate, bearing row of spinules on posterior margin and 3 terminal elements; medial element longest; inner and medial element with bilateral spinulation; outer element shortest, with serrated margins.

Leg 5 (Fig. 8C) 2-segmented; protopodal segment with naked outer seta; exopodal segment bearing 2 terminal setal elements and patch of spinules; spinules becoming more elongate towards outer posterior margin; both setal elements with bilateral spinulation; outer element twice as long as inner. Leg 6 not seen.

### Remarks

There are 8 nominal species of *Unicolax*: *U. anonymous* (Vervoort, 1965) Cressey & Cressey, 1980; *U. ciliatus* Cressey & Cressey, 1980; *U. collateralis* Cressey & Cressey, 1980; *U. longispinus* Lin & Ho, 2006; *U. mycterobius* (Vervoort, 1965) Cressey & Cressey, 1980; *U. quadrispinulus* Lin & Ho, 2006; *U. reductus* Cressey & Cressey, 1980, and *U. longicrus* n. sp. The most recent key to *Unicolax* is by Lin & Ho (2006) and includes all species but *U. longicrus* n. sp.

The new species is distinguished from *U. reductus* in its lack of conspicuous dorsolateral aliform expansions of the second pedigerous somite. It further differs in having a leg 4 exopod formula of II, I, 4 rather than II, I, 3. The new species can be differentiated from *U. ciliatus*, *U. collateralis*, *U. mycterobius*, *U. longispinus,* and *U. quadrispinulus* by its possession of a leg 4 exopod with the setal formula II, I, 4 rather than II, I, 5. The new species resembles *U. anonymous* in this feature, but differs in its possession of a leg 5 that is relatively longer and less wide. The setation of the fifth leg also differs, whereas *U. anonymous* possesses inner and outer distal spines on leg 5 that are approximately the same length, those of the new species are relatively longer and asymmetrical (outer spine approximately 40% the length of terminal seta, inner spine approximately 50% the length of terminal seta). *Unicolax longicrus* n. sp. can also be differentiated from *U. anonymous* in its possession of a leg 4 with much more elongate endopodal segments 2 and 3. The new species further differs from *U. anonymous*, *U. collateralis*, *U. reductus,* and, to a lesser degree, *U. ciliatus*, in its possession of outer spines on the exopodal segments, particularly of legs 3 and 4, with smaller, less robust serrations on their margins.

The new species is most similar to *U. quadrispinulus*, the only other species of *Unicolax* known to parasitize a host species of the genus *Sillago.* Both species possess four spines on the distal exopodal segment of leg 1. The new species differs from *U. quadrispinulus* in a number of features. There are numerous differences in leg 4 between the new species and *U. quadrispinulus*: the distal exopodal segment is II, I, 4 in the new species and II, I, 5 in *U. quadrispinulus*; the setal element on the second endopodal segment is much shorter and more spine-like in the new species, extending only to the midline of the distal endopodal segment, while in *U. quadrispinulus* this element is a plumose seta that extends well beyond the end of the ramus and is more than 1.5× the length of distal segment. The new species is unusual among species of *Unicolax* in possessing elongate endopodal segments in leg 4: in the new species the second endopodal segment of leg 4 has a length:width ratio of 2.5 vs. 1.6 in *U. quadrispinulus* and the distal segment has a length:width ratio of 3.8 vs. 2.4 in *U. quadrispinulus*. In addition to the differences in leg 4, the lateral terminal spines of leg 5 in *U. quadrispinulus* are the same length, whereas in the new species the inner distal spine is 30% longer than the outer distal spine. The spinules on the antenna of the new species are also larger and less densely arrayed relative to those on *U. quadrispinulus* (see Lin & Ho, 2006: fig. 13B). Furthermore, the terminal spine of the distal endopodal segment of leg 3 is longer than the segment itself in *U. quadrispinulus* while the terminal spine of the new species is shorter than the segment and more blunt.

### *Unicolax* host associations and biogeography

The hosts and localities of all known species of *Unicolax* are summarized in Table 4*.* The 8 species of *Unicolax* have collectively been reported 83 times. The genus parasitizes the nasal cavity of at least 2 orders of fish. Six of 8 known species of *Unicolax* parasitize fish of the order Scombriformes. *Unicolax quadrispinulus* and *U. longricrus* parasitize species of *Sillago* (family Sillaginidae, Order *incertae sedis* in Eupercaria). Three fish families are known to host *Unicolax*. Five species of *Unicolax* are known to parasitize the Scombridae, 2 the Sillaginidae, and *U. longispinus* the Centrolophidae.

**Table 4:** *Unicolax* species hosts and localities. Records in bold indicate type hosts and localities.

**Species**	**Host**	**Host family**	**Host order**	**Locality**	**Marine ecoregion**	**Source**
*U*. *anonymus* (Vervoort, 1965) Cressey & Cressey, 1980	***Euthynnus alletteratus***	Scombridae	Scombriformes	**Abidjan, Côte dTvoire, Gulf of Guinea**	Tropical Atlantic	Vervoort (1965)
	*Euthynnus alletteratus*	Scombridae	Scombriformes	Ghana	Tropical Atlantic	Cressey & Cressey (1980)
	*Euthynnus alletteratus*	Scombridae	Scombriformes	Gulf of Mexico	Tropical Atlantic	Cressey & Cressey (1980)
*U. ciliatus* Cressey & Cressey, 1980	***Scomberomorus plurilineatus***	Scombridae	Scombriformes	**Zanzibar Channel**	Western Indo Pacific	Cressey & Cressey (1980)
	*Scomberomorus commerson*	Scombridae	Scombriformes	Batavia, Java	Central Indo Pacific	Cressey & Cressey (1980)
	*Scomberomorus commerson*	Scombridae	Scombriformes	Hong Kong	Central Indo Pacific	Cressey & Cressey (1980)
	*Scomberomorus commerson*	Scombridae	Scombriformes	Mi-Tuo fishing port, Taiwan	Central Indo Pacific	Lin & Ho (2006)
	*Scomberomorus commerson*	Scombridae	Scombriformes	Philippines	Central Indo Pacific	Cressey & Cressey (1980)
	*Scomberomorus commerson*	Scombridae	Scombriformes	Cochin, India	Western Indo Pacific	Cressey & Cressey (1980)
	*Scomberomorus commerson*	Scombridae	Scombriformes	Madagascar	Western Indo Pacific	Cressey & Cressey (1980)
	*Scomberomorus commerson*	Scombridae	Scombriformes	Phuket, Thailand	Western Indo Pacific	Cressey & Cressey (1980)
	*Scomberomorus commerson*	Scombridae	Scombriformes	Travancore, India	Western Indo Pacific	Cressey & Cressey (1980)
	*Scomberomorus guttatus*	Scombridae	Scombriformes	Batavia, Java	Central Indo Pacific	Cressey & Cressey (1980)
	*Scomberomorus guttatus*	Scombridae	Scombriformes	Hong Kong	Central Indo Pacific	Cressey & Cressey (1980)
	*Scomberomorus guttatus*	Scombridae	Scombriformes	Singapore	Central Indo Pacific	Cressey & Cressey (1980)
	*Scomberomorus guttatus*	Scombridae	Scombriformes	Calicut, India	Western Indo Pacific	Cressey & Cressey (1980)
	*Scomberomorus guttatus*	Scombridae	Scombriformes	Padang, Sumatra	Western Indo Pacific	Cressey & Cressey (1980)
	*Scomberomorus guttatus*	Scombridae	Scombriformes	Phuket, Thailand	Western Indo Pacific	Cressey & Cressey (1980)
	*Scomberomorus guttatus*	Scombridae	Scombriformes	China	NA	Cressey & Cressey (1980)
	*Scomberomorus niphonius*	Scombridae	Scombriformes	Korea	Temperate Northern Pacific	Cressey & Cressey (1980)
	*Scomberomorus niphonius*	Scombridae	Scombriformes	China	NA	Cressey & Cressey (1980)
	*Scomberomorus semifasciatus*	Scombridae	Scombriformes	New Guinea	Central Indo Pacific	Cressey & Cressey (1980)
	*Scomberomorus tritor*	Scombridae	Scombriformes	Ghana	Tropical Atlantic	Cressey & Cressey (1980)
	*Scomberomorus tritor*	Scombridae	Scombriformes	Lagos, Nigeria	Tropical Atlantic	Cressey & Cressey (1980)
	*Scomberomorus tritor*	Scombridae	Scombriformes	Liberia	Tropical Atlantic	Cressey & Cressey (1980)
*U. collateralis* Cressey & Cressey, 1980	***Euthynnus alletteratus***	Scombridae	Scombriformes	**Saint George Bay, Lebanon**	Temperate Northern Atlantic	Cressey & Cressey (1980)
	*Auxis* sp.	Scombridae	Scombriformes	Hong Kong	Central Indo Pacific	Cressey & Cressey (1980)
	*Auxis* sp.	Scombridae	Scombriformes	Philippines	Central Indo Pacific	Cressey & Cressey (1980)
	*Auxis* sp.	Scombridae	Scombriformes	Hawaii	Eastern Indo Pacific	Cressey & Cressey (1980)
	*Auxis* sp.	Scombridae	Scombriformes	Saint George Bay, Lebanon	Temperate Northern Atlantic	Cressey & Cressey (1980)
	*Auxis* sp.	Scombridae	Scombriformes	Woods Hole, Massachusetts	Temperate Northern Atlantic	Cressey & Cressey (1980)
	*Auxis* sp.	Scombridae	Scombriformes	Chusan, China	Temperate Northern Pacific	Cressey & Cressey (1980)
	*Auxis* sp.	Scombridae	Scombriformes	Japan	Temperate Northern Pacific	Cressey & Cressey (1980)
	*Auxis* sp.	Scombridae	Scombriformes	Ghardaqa, Egypt	Western Indo Pacific	Cressey & Cressey (1980)
	*Cybiosarda elegans*	Scombridae	Scombriformes	Brisbane, Australia	Central Indo Pacific	Cressey & Cressey (1980)
	*Euthynnus alletteratus*	Scombridae	Scombriformes	Caribbean 9°11′N, 77°50′W	Tropical Atlantic	Cressey & Cressey (1980)
	*Euthynnus alletteratus*	Scombridae	Scombriformes	Brazil	Tropical Atlantic	Cressey & Cressey (1980)
	*Euthynnus alletteratus*	Scombridae	Scombriformes	Southeastern Iberia	Temperate Northern Atlantic	Mele et al. (2016)
	*Euthynnus alletteratus*	Scombridae	Scombriformes	Saint George Bay, Lebanon	Temperate Northern Atlantic	Cressey & Cressey (1980)
	*Euthynnus lineatus*	Scombridae	Scombriformes	Galapagos	Tropical Eastern Pacific	Cressey & Cressey (1980)
	*Euthynnus lineatus*	Scombridae	Scombriformes	Lower California	Temperate Northern Pacific	Cressey & Cressey (1980)
	*Euthynnus lineatus*	Scombridae	Scombriformes	Mexico (Pacific)	Tropical Eastern Pacific	Cressey & Cressey (1980)
	*Euthynnus lineatus*	Scombridae	Scombriformes	Costa Rica (Pacific)	Tropical Eastern Pacific	Cressey & Cressey (1980)
	*Euthynnus affinis*	Scombridae	Scombriformes	Brisbane, Australia	Central Indo Pacific	Cressey & Cressey (1980)
	*Euthynnus affinis*	Scombridae	Scombriformes	Formosa	Central Indo Pacific	Cressey & Cressey (1980)
	*Euthynnus affinis*	Scombridae	Scombriformes	Gulf of Thailand	Central Indo Pacific	Cressey & Cressey (1980)
	*Euthynnus affinis*	Scombridae	Scombriformes	Hong Kong	Central Indo Pacific	Cressey & Cressey (1980)
	*Euthynnus affinis*	Scombridae	Scombriformes	Java	Central Indo Pacific	Cressey & Cressey (1980)
	*Euthynnus affinis*	Scombridae	Scombriformes	Palau	Central Indo Pacific	Cressey & Cressey (1980)
	*Euthynnus affinis*	Scombridae	Scombriformes	Phillipines	Central Indo Pacific	Cressey & Cressey (1980)
	*Euthynnus affinis*	Scombridae	Scombriformes	Okinawa	Temperate Northern Pacific	Cressey & Cressey (1980)
	*Euthynnus affinis*	Scombridae	Scombriformes	Tokyo	Temperate Northern Pacific	Cressey & Cressey (1980)
	*Euthynnus affinis*	Scombridae	Scombriformes	Arabian Sea	Western Indo Pacific	Cressey & Cressey (1980)
	*Euthynnus affinis*	Scombridae	Scombriformes	Madagascar	Western Indo Pacific	Cressey & Cressey (1980)
	*Euthynnus affinis*	Scombridae	Scombriformes	Mozambique	Western Indo Pacific	Cressey & Cressey (1980)
	*Euthynnus affinis*	Scombridae	Scombriformes	Seychelles	Western Indo Pacific	Cressey & Cressey (1980)
	*Euthynnus affinis*	Scombridae	Scombriformes	Elat, Israel	Western Indo Pacific	Cressey & Cressey (1980)
	*Orcynopsis unicolor*	Scombridae	Scombriformes	Saint George Bay, Lebanon	Temperate Northern Atlantic	Cressey & Cressey (1980)
	*Sarda australis*	Scombridae	Scombriformes	New South Wales, Australia	Temperate Australasia	Cressey & Cressey (1980)
	*Sarda orientalis*	Scombridae	Scombriformes	Durban, South Africa	Temperate Southern Africa	Cressey & Cressey (1980)
	*Sarda orientalis*	Scombridae	Scombriformes	Pearl Island, Panama	Tropical Eastern Pacific	Cressey & Cressey (1980)
*U. longicrus* n. sp.	***Sillgao maculata***	Sillaginidae	Order *incertae sedis* in Eupercaria	**Moreton Bay, Queensland, Australia 27°26′S, 153°24′E**	Central Indo Pacific	Present study
	*Sillago ciliata*	Sillaginidae	Order *incertae sedis* in Eupercaria	Moreton Bay, Queensland, Australia 27°22′S, 153°13′E	Central Indo Pacific	Present study
*U. longispinus* Lin & Ho, 2006	***Psenopsis anomala***	Centrolophidae	Scombriformes	**Hsing-Dah fishing port, Taiwan (Kaohsiung County)**	Central Indo Pacific	Lin & Ho (2006)
	*Psenopsis anomala*	Centrolophidae	Scombriformes	Dong-Shih fishing port, Taiwan	Central Indo Pacific	Lin & Ho (2006)
*U*. *mycterobius* (Vervoort, 1965) Cressey & Cressey, 1980	***Auxis thazard***	Scombridae	Scombriformes	**Gulf of Guinea, off Abidjan, Côte dTvoire**	Tropical Atlantic	Vervoort (1965)
	*Auxis rochei*	Scombridae	Scombriformes	Strait of Gibraltar	Temperate Northern Atlantic	Mele et al. (2015)
	*Auxis* sp.	Scombridae	Scombriformes	Formosa	Central Indo Pacific	Cressey & Cressey (1980)
	*Auxis* sp.	Scombridae	Scombriformes	Luzon, Phillipines	Central Indo Pacific	Cressey & Cressey (1980)
	*Auxis* sp.	Scombridae	Scombriformes	Hawaii	Eastern Indo Pacific	Cressey & Cressey (1980)
	*Auxis* sp.	Scombridae	Scombriformes	New South Wales, Australia	Temperate Australasia	Cressey & Cressey (1980)
	*Auxis* sp.	Scombridae	Scombriformes	Massachusetts	Temperate Northern Atlantic	Cressey & Cressey (1980)
	*Auxis* sp.	Scombridae	Scombriformes	Tokyo	Temperate Northern Pacific	Cressey & Cressey (1980)
	*Auxis* sp.	Scombridae	Scombriformes	Ghardaqa, Egypt	Western Indo Pacific	Cressey & Cressey (1980)
	*Euthynnus alletteratus*	Scombridae	Scombriformes	Saint George Bay, Lebanon	Temperate Northern Atlantic	Cressey & Cressey (1980)
	*Euthynnus alletteratus*	Scombridae	Scombriformes	Pensacola, Florida	Tropical Atlantic	Cressey & Cressey (1980)
	*Euthynnus affinis*	Scombridae	Scombriformes	Kagoshima, Japan	Temperate Northern Pacific	Cressey & Cressey (1980)
	*Euthynnus affinis*	Scombridae	Scombriformes	Tokyo	Temperate Northern Pacific	Cressey & Cressey (1980)
*U. quadrispinulus* Lin & Ho, 2006	***Sillago sihama***	Sillaginidae	Order *incertae sedis* in Eupercaria	**Hsing-Dah fishing port, Taiwan (Kaohsiung County)**	Central Indo Pacific	Lin & Ho (2006)
	*Sillago sihama*	Sillaginidae	Order *incertae sedis* in Eupercaria	Dong-Shih fishing port, Taiwan	Central Indo Pacific	Lin & Ho (2006)
*U. reductus* Cressey & Cressey, 1980	***Katsuwonus pelamis***	Scombridae	Scombriformes	**New South Wales**	Temperate Australasia	Cressey & Cressey (1980)
	*Katsuwonus pelamis*	Scombridae	Scombriformes	Tahiti	Eastern Indo Pacific	Cressey & Cressey (1980)
**	*Katsuwonus pelamis*	Scombridae	Scombriformes	Japan	Temperate Northern Pacific	Cressey & Cressey (1980)

Species of *Unicolax* are known from 9 of the 12 marine realms established by Spalding et al. (2007). Of the 83 reports of *Unicolax* reviewed*,* 26 (31%) come from the Central Indo-Pacific realm, followed by 15 (18%) from the Western Indo-Pacific, 10 each (12%) in the Temperate Northern Pacific and Tropical Atlantic, and 9 or fewer in the following 5 realms: Temperate Northern Atlantic (*n* = 9; 11%), Tropical Eastern Pacific (*n* = 4; 5%), Eastern Indo-Pacific (*n* = 3; 4%), Temperate Australasia (*n* = 3; 4%), and Temperate Southern Africa (*n* = 1; 1%) . There are currently no reports of *Unicolax* from the Arctic, Temperate South America, or Southern Ocean realms. Two reports of *U. ciliatus* by Cressey & Cressey (1980) could not be assigned to a realm. The locality was given only as “China” (Cressey & Cressey, 1980 pg. 15), and given that the coast of China spans the Central Indo-Pacific and Temperate Northern Pacific realms, a biogeographic realm could not be unambiguously assigned to these records.

## Discussion

Current reports suggest *Nothobomolochus* has a predominately tropical distribution, with 86 reports from tropical ecoregions and 24 reports from temperate regions. The west coast of North America and the east and west coasts of South America remain largely unexplored for species of *Nothobomolochus*. Given the diversity of potential hosts there, we suspect many new species remain to be described from beloniform, scombriform, and clupeiform fishes in these waters.

Of the 39 species of *Nothobomolochus*, 25 have been reported a single time, only from the host from which they were described. By far the most widely reported species of *Nothobomolochus* is *N. gibber*, which has been reported 28 times from beloniform fish of the families Belonidae and Hemiramphidae. The global distribution of this species, ranging from the Mediterranean Sea to Ascension Island in the Atlantic and a variety of localities spanning the Indian and Pacific Oceans calls into question whether these specimens are, in fact, conspecific. Because of the limited nature of host reports for species of *Nothobomolochus*, few negative host data available, and questionable conspecificity of specimens reported from a variety of hosts and geographic regions, a precise measure of the host specificity of the genus remains elusive.

Most reports of *Unicolax* come from host species of the family Scombridae. The report of *U. longispinus* from *Psenopsis anomala* (Temminck & Schlegel, 1844) (Centrolophidae), the first species reported from a non-scombrid host, is not particularly surprising given that the Centrolophidae are closely related to the Scombridae, an affinity supported by mulitlocus phylogenetic analyses (Betancur et al., 2017). Both families, in fact, are now recognized as members of the order Scombriformes (Betancur et al., 2017). However, the discovery of *U. quadrispinulus* and *U. longicrus* parasitizing species of *Sillago* is unexpected given the distant phylogenetic relationship of these hosts with the Scombridae (see Betancur et al., 2017 fig. 2). This suggests that, not only are other scombrids and related families (i.e., Amarsipidae, Ariommatidae, Arripidae, Bramidae, Caristiidae, Chiasmodontidae, Gempylidae, Icosteidae, Nomeidae, Pomatomidae, Scombropidae, Scombrolabracidae, Stromateidae, Tetragonuridae, and Trichiuridae) candidate hosts of *Unicolax*, but also that much of the Eupercaria, the largest series of fishes, containing over 6,000 species in 161 families and at least 17 orders, are potential hosts.

The fact that most known hosts of *Unicolax* are widely harvested, economically important fish species is likely a reflection of sampling bias rather than true host distribution. Both the inaccessible microhabitat (i.e., the nasal sinuses) parasitized by these copepods and their small size (0.6–3 mm) has contributed to the slow discovery of species of *Unicolax*. Given the phylogenetic diversity of fish species hosting members of *Unicolax*, we predict that careful observation of the nasal sinuses of marine fish will reveal numerous additional scombriform and eupercarian fish host species of *Unicolax*, many of which are likely new to science. It is likely that diversity of this genus is substantially higher than current records suggest, and this also likely applies to other copepod genera that predominately inhabit fish nostrils.

Five genera of bomolochids are known to live almost exclusively in the nostrils of teleosts: *Acanthocolax* Vervoort, 1969*, Ceratocolax* Vervoort, 1965*, Naricolax* Ho, Do & Kasahara, 1983*, Tegobomolochus* Izawa, 1976, and *Unicolax*. These genera share a number of morphological similarities and are thought to be closely related (Huys et al., 2012). It is interesting to consider the potential functional significance of shared morphological features in this group. For instance, the second leg of *Ceratocolax*, *Naricolax, Unicolax*, and *Tegobomolochus* have flattened endopods, and Huys et al. (2012) proposed this modification may help seal the suction cup formed by the ventral cephalothorax, a process documented in other parasitic copepods (e.g., leg 3 of Caligidae by Kabata & Hewitt (1971)). Similarly, males of *Naricolax, Unicolax,* and *Tegobomolochus* possess a flattened leg 1, which may assist in sealing the cephalothoracic suction cup.

Other structures have arisen in a number of nostril inhabiting copepods. A pincer-like structure arising from projections of anterior cephalothorax and dorsal projections of the antennae is present in *Acanthocolax, Ceratocolax*, and *Tegobomolochus* (Huys et al., 2012). There may be some evidence of this, albeit to a lesser degree, in species of *Unicolax*. Vervoort (1965) shows *U. anonymous* to have a protrusion at the anterior margin of the cephalothorax (fig. 1) and Cressey & Cressey (1980) illustrated a similar protrusion in *U. collateralis* (see fig. 9A). Perhaps a pincer-like structure is formed between this anterior protrusion of the cephalothorax and the modified spine-like seta of the antennule of these species, which may provide a functional explanation for the heavily sclerotized fourth seta of the antennule in *Unicolax*. It would be interesting to explore if these pincer-like structures are used for attachment in the nostril, perhaps to the lamellae of the olfactory rosette. A number of nostril inhabiting copepods have also developed dorsal extensions on their body somites (e.g., *Ceratocolax, Tegobomolochus*, *U. anonymous* [see Vervoort, 1965 fig. 1], and *U. reductus* [Cressey & Cressey, 1980 fig. 23a]). Given that dorsal extensions appear to have arisen multiple times in nostril-inhabiting copepods, it is possible that they have functional significance; for instance, they may reduce shearing forces on the copepods or enable them to wedge themselves in small cavities of the nasal passage.

There is considerable variation in the ornamentation on *Unicolax* appendages. In particular, ornamentation on the margins of spines on legs 2–4 varies from relatively few, robust serrations in *U. anonymous, U. collateralis*, and *U. reductus*, to numerous fine serrations along the margins of the spines in *U. ciliatus*, *U. longicrus, U. longispinus, U. mycterobius,* and *U. quadrispinulus*. The inner seta on leg 4 also varies from a typical plumose seta, as seen in *U. quadrispinulus*, to a more spine-like element in most other species of *Unicolax*; this element is highly reduced to a short spine-like element in *U. reductus*. We recommend researchers pay careful attention to the ornamentation of setal elements in species of *Unicolax*, as these are likely to be useful taxonomic characters.

##  Supplemental Information

10.7717/peerj.6858/supp-1 Table S1 *Nothobomolochus* species hosts and localities Records in bold indicate type hosts and localities.

10.7717/peerj.6858/supp-2 Table S2 *Unicolax* species hosts and localities Records in bold indicate type hosts and localities.

## Additional Information and Declarations

### Field Study Permissions

The following information was supplied relating to field study approvals (i.e., approving body and any reference numbers): Australian Department of Agriculture, Fisheries, and Forestry provided permit number 187264.

### Data Availability

The following information was supplied regarding data availability: The raw data used in the beogeography and host association summaries are available in Tables S1 and S2. Museum accession numbers are: *Nothobomolochus johndaveorum* n. sp. Holotype female (QM W29438) and 10 female paratypes (2 paratypes QM W29439; 4 paratypes NHMUK 2018.194–2018.197; 4 paratypes USNM 1532298–1532300). *Unicolax longicrus* n. sp. Holotype female (QM W29434) and 5 female paratypes (1 paratype QM W29435; 2 paratypes NHMUK 2018.198–2018.199; 2 paratypes USNM 1532294–1532295). Allotype male (QM W29436) and 4 male paratypes (2 paratypes NHMUK 2018.200–2018.201; 2 paratypes USNM 1532296–1532297).

### New Species Registration

The following information was supplied regarding the registration of a newly described species: Publication LSID: urn:lsid:zoobank.org:pub:E8663AB9-EF47-4382-8096-C45B10A879A7. *Nothobomolochus johndaveorum* LSID: urn:lsid:zoobank.org:act:D5F768DE-686A-4B00-B241-8BBA061BF4A9. *Unicolax longicrus* LSID: urn:lsid:zoobank.org:act:0E24C5F0-29C1-49C9-830D-7124D27D3FAE.
